# Genome-wide mapping of histone modifications during axenic growth in two species of *Leptosphaeria maculans* showing contrasting genomic organization

**DOI:** 10.1007/s10577-021-09658-1

**Published:** 2021-05-21

**Authors:** Jessica L. Soyer, Colin Clairet, Elise J. Gay, Nicolas Lapalu, Thierry Rouxel, Eva H. Stukenbrock, Isabelle Fudal

**Affiliations:** 1 Université Paris-Saclay, INRAE, AgroParisTech, UMR BIOGER, 78850 Thiverval-Grignon, France; 2 Max Planck Institute for Evolutionary Biology, August-Thienemann-Str. 2, 24306 Plön, Germany; 3 Christian-Albrechts University of Kiel, Am Botanischen Garten 1-9, 24118 Kiel, Germany

**Keywords:** *Leptosphaeria maculans*, ChIP-seq, comparative epigenomics, heterochromatin, effectors, host-pathogen interaction

## Abstract

*Leptosphaeria maculans* ‘brassicae’ (Lmb) and *Leptosphaeria maculans* ‘lepidii’ (Lml) are closely related phytopathogenic species that exhibit a large macrosynteny but contrasting genome structure. Lmb has more than 30% of repeats clustered in large repeat-rich regions, while the Lml genome has only a small amount of evenly distributed repeats. Repeat-rich regions of Lmb are enriched in effector genes, expressed during plant infection. The distinct genome structures of Lmb and Lml provide an excellent model for comparing the organization of pathogenicity genes in relation to the chromatin landscape in two closely related phytopathogenic fungi. Here, we performed chromatin immunoprecipitation (ChIP) during axenic culture, targeting histone modifications typical for heterochromatin or euchromatin, combined with transcriptomic analysis to analyze the influence of chromatin organization on gene expression. In both species, we found that facultative heterochromatin is enriched with genes lacking functional annotation, including numerous effector and species-specific genes. Notably, orthologous genes located in H3K27me3 domains are enriched with effector genes. Compared to other fungal species, including Lml, Lmb is distinct in having large H3K9me3 domains associated with repeat-rich regions that contain numerous species-specific effector genes. Discovery of these two distinctive heterochromatin landscapes now raises questions about their involvement in the regulation of pathogenicity, the dynamics of these domains during plant infection and the selective advantage to the fungus to host effector genes in H3K9me3 or H3K27me3 domains.

**Supplementary Information:**

The online version contains supplementary material available at 10.1007/s10577-021-09658-1.

## Introduction

Each year, hundreds of millions of tons of agricultural crops are devastated by plant pathogenic fungi or made unfit for consumption due to contamination by mycotoxins (Fisher et al. 2018). Management strategies to control fungal infection mainly involve chemical control or breeding for naturally resistant crop cultivars. However, fungal plant pathogens have proven capable of rapidly evolving resistance against fungicides (Fisher et al. 2018) and to overcome specific plant resistance genes within a few years (for instance in *Leptosphaeria maculans*; Rouxel and Balesdent 2017), emphasizing the need for improved control methods.

Understanding the determinants of the extreme adaptive abilities of fungal plant pathogens is a critical issue for the development of effective and sustainable control methods. In that respect, comparative and population genomic analyses have provided new insights into the evolutionary dynamics of fungal plant pathogens (e.g. Stukenbrock et al. 2011; Grandaubert et al. 2014; Sánchez-Vallet et al. 2018). Notably, transposable elements (TE) have been shown to play a crucial role in shaping the genome structure of plant pathogenic fungi (Ohm et al. 2012; Grandaubert et al. 2014; Möller and Stukenbrock 2017; Seidl and Thomma 2017). TEs are often organized in clusters, compartmentalizing the genome into gene-rich regions and TE-rich regions (e.g. in *L. maculans* and in *Mycosphaerella fijiensis*; Rouxel et al. 2011; Ohm et al. 2012; Grandaubert et al. 2014). While the gene density in TE-rich regions is low, genes located in these regions have been shown to evolve faster than genes located in TE-poor regions (Croll and McDonald 2012; de Jonge et al. 2013). Interestingly, rapidly evolving genes in TE-rich regions have, in many cases, been identified as genes involved in niche adaptation and notably effector genes. Effectors are considered as key elements of pathogenesis, allowing pathogens to circumvent host recognition, impede defence reactions and facilitate host invasion. Effectors are mainly secreted proteins (called proteinaceous effectors), but can also be secondary metabolites (called metabolic effectors) or small RNAs (Lo Presti et al. 2015; Collemare et al. 2019; Rocafort et al. 2020). Plants have evolved strategies to recognize and counteract effectors, exposing them to a strong selection pressure by the host immune system (Stergiopoulos et al. 2007; Chuma et al. 2011; Rouxel and Balesdent 2017). Indeed, in the course of the co-evolution between a pathogen and its host, the host has developed an active immune system allowing the direct or indirect recognition of some effector molecules, to activate defence responses, often involving a local cell death, called the hypersensitive response. Effectors that can be recognized by the host are called avirulence proteins (Jones and Dangl 2006).

*Leptosphaeria maculans* ‘brassicae’ (hereinafter referred to as Lmb) belongs to the Dothideomycete class of Ascomycete fungi and is responsible for causing stem canker of oilseed rape (*Brassica napus*). Lmb displays a complex, hemibiotrophic life cycle, during which it alternates between different nutritional modes on its host plant. It causes necrosis on different plant organs: leaves, more rarely seedpods and the base of the stem, causing lodging of the plant and yield losses (Rouxel and Balesdent 2005). The most efficient method of disease control relies on the use of major resistance genes present in oilseed rape and other Brassica species. Although efficient, this control method is not sustainable, as Lmb is able to ‘break down’ novel sources of genetic resistance rapidly (Rouxel and Balesdent 2017). One-third of the Lmb genome is made of TE-rich regions. These regions are enriched with putative effector genes and include all currently known avirulence genes. These avirulence genes are highly expressed in the first 7 days of leaf and cotyledon infection (Gout et al. 2006; Fudal et al. 2007; Parlange et al. 2009; Balesdent et al. 2013; Van de Wouw et al. 2014; Ghanbarnia et al. 2015, 2018; Plissonneau et al. 2016, 2018; Petit-Houdenot et al. 2019; Neik et al. 2020). Another set of putative proteinaceous effector genes are located in gene-rich regions of the genome and are specifically expressed during stem infection (Gervais et al. 2017).

In eukaryotic cells, chromatin can adopt different conformational states directly influencing gene expression: gene-rich euchromatin, sheltering constitutively expressed genes, and gene-poor heterochromatin, in which genes are silent. The different chromatin states are characterized by different post-translational modifications of histones around which DNA is wrapped. Typically, heterochromatin is enriched in the trimethylation of the lysine 9 of histone H3 (H3K9me3) and lysine 27 (H3K27me3) while euchromatin is enriched in the di- (or tri-) methylation of the lysine 4 of histone H3 (H3K4me2); (Mikkelsen et al. 2007; Jamieson et al. 2013). In fungal plant pathogens or endophytes, evidence is accumulating that transcriptional reprogramming of effector genes (either proteinaceous or metabolic) is tightly controlled by chromatin-based regulatory mechanisms (for example in *Fusarium graminearum*, *Epichloe festucae*, Lmb and *Zymoseptoria tritici*; Connolly et al. 2013; Chujo and Scott 2014; Soyer et al. 2014, 2015a, 2019; Lukito et al. 2020; Meile et al. 2020). In Lmb, the location of avirulence genes in TE-rich regions has an influence on their evolution under selection pressure (Rouxel et al. 2011; Daverdin et al. 2012) and plays a role in regulating their expression during axenic culture via deposition of H3K9me3 (Soyer et al. 2014).

Lmb belongs to the *L. maculans*/*Leptosphaeria biglobosa* species complex, comprising species with different host specialization and genome organization (Grandaubert et al. 2014). Within the species complex, the *L. maculans* species infecting oilseed rape, Lmb, is the only one having large regions of its genome enriched with TEs while other genomes have a low TE content (~3–15% compared to more than 30% TEs in Lmb; Rouxel et al. 2011; Grandaubert et al. 2014; Dutreux et al. 2018). For example, the genome of the species that is most closely related to Lmb, *Leptosphaeria maculans* ‘lepidii’ (hereinafter referred to as Lml), which infects crucifers such as *Lepidium sativum*, and to a lesser extent *Camelina sativa* and *Brassica rapa* (Petrie 1969), has a low TE content (3% of TEs; Grandaubert et al. 2014). Interestingly, the genomes of Lmb and Lml show a high level of macrosynteny, with only a few intra-chromosomal inversions, but differ in their TE content with Lmb having undergone a massive TE expansion 5 million years ago corresponding to the speciation date (Grandaubert et al. 2014). Invasion of TEs in the genome of Lmb has shaped its genome, with alternating TE-rich regions and gene-rich regions. In contrast, the Lml genome shows a homogeneous TE distribution along the chromosomes (Grandaubert et al. 2014).

The distinct genome organization shown by Lmb and Lml thus provides us with a model of choice for comparing epigenomic organization in two closely related phytopathogenic fungi, to determine the genomic location of pathogenicity/effector genes in relation to the chromatin landscape and the influence of chromatin structure on gene expression. Comparative epigenomic analyses, at the intra- or inter-species levels, are very sparse and this remains an underexplored field of study, at least in fungi (Zhang et al. 2018; Feurtey et al. 2019). We present here the first comparative epigenomic analysis of both euchromatin and heterochromatin marks in two closely related phytopathogenic fungi. We first performed ChIP-seq and RNA-seq during axenic culture to compare the distribution of three histone modifications, H3K4me2, H3K9me3 and H3K27me3 and then investigated whether chromatin organization is conserved between Lmb and Lml by assessing whether orthologous genes, and pathogenicity-related genes, are located in similar chromatin domains in each species. Lastly, we assessed the influence of the chromatin landscape on gene expression during axenic growth, focusing mostly on pathogenicity-related genes that may be species specific.

## Materials and methods

### Fungal isolates

The isolate v23.1.3 of *L. maculans* ‘brassicae’ and the isolate IBCN84 of *L. maculans* ‘lepidii’ were used throughout the analyses (Rouxel et al. 2011; Grandaubert et al. 2014). Fungal cultures were maintained as described previously (Ansan-Melayah et al. 1995). For chromatin immunoprecipitation and transcriptomic analyses performed during in vitro growth, mycelium of Lmb and Lml were grown on V8 agar medium at 25 °C for 7 days. Then 10 plugs of mycelium were inoculated into 100 ml of Fries liquid medium in a Roux bottle. Mycelia were harvested after growing for 7 days at 25 °C, filtered and washed thoroughly with distilled water and immediately placed in liquid nitrogen until further used. ChIP experiments were performed on fresh material.

### Chromatin immunoprecipitation and high-throughput sequencing

ChIP was performed from freshly harvested mycelium grown in Fries liquid culture, as described in Soyer et al. (2015b), with minor modifications. ChIP was performed on native material (without crosslinking) using antibodies targeting histone modifications H3K4me2 (Merck ref. 07-030), H3K9me3 (Active Motif, Carlsbad, CA, USA; ref. 39,161) or H3K27me3 (Active Motif, Carlsbad, CA, USA; ref. 39155). Three different ChIPs (i.e. three biological replicates) were performed for each of the histone modifications. Libraries were prepared from all biological replicates, individually, according to the Illumina TruSeq protocol ‘Ultra Low Input DNA library’. Libraries were sequenced with an Illumina HiSeq 2000 genome analyzer at the Max Planck Genome Centre Cologne, Germany (https://mpgc.mpipz.mpg.de/home/). Sequencing data are available under the GEO accession number GSE150127.

### Analysis of ChIP-seq data and identification of significantly enriched domains

Analysis of ChIP-seq datasets was performed as described in Schotanus et al. (2015). Quality of Illumina reads was analyzed using FastQC (https://www.bioinformatics.babraham.ac.uk/projects/fastqc/). Based on results of this analysis, 10 bp were trimmed from the 5′ end. Processed reads were mapped on the reference genome of Lmb (Dutreux et al. 2018) or Lml (Grandaubert et al. 2014) using Bowtie2 (Langmead and Salzberg 2012) with default parameters (Supplementary Table 1). Peak calling analysis was performed on each ChIP sequencing dataset, to identify significantly enriched domains for either H3K4me2, H3K9me3 or H3K27me3, using RSEG (Song and Smith 2011). A domain was considered if identified in at least two out of the three biological replicates. The Integrative Genome Viewer (Thorvaldsdóttir et al. 2013) was used to visualize location of each domain along genomes of Lmb and Lml according to other genome features (gene annotation, TE annotation, GC content). Coverage of the histone modifications was assessed in 10-kb non-overlapping sliding windows along the supercontigs and correlation analyses, using Kendall’s *Ƭ* correlation coefficient, were performed between biological replicates to check for reproducibility (Supplementary Tables 2 and 3).

### RNA extraction, RNA-sequencing and expression analysis

Total RNA was extracted from mycelium grown for 1 week in Fries liquid medium as previously described (Fudal et al. 2007). The NEBNext Ultra Directional RNA Library Prep Kit for Illumina (cat. # E7420L New England BioLabs) was used to prepare RNA-seq libraries and sequencing was performed on a HiSeq 4000 Illumina genome analyzer using 50 paired-end reads. Raw reads were then pre-processed with Trimmomatic (Bolger et al. 2014) to remove short reads (<30 bp) and eliminate sequencing adaptors. Cleaned reads were then mapped against each genome using STAR with default parameters (Dobin and Gingeras 2015; Supplementary Table 1). Gene expression was evaluated using the rpkm_count function of EdgeR (Robinson et al. 2010). Genes with RPKM ≥ 2 were considered expressed. Sequencing data are available under the GEO accession number GSE150127.

### Identification of orthologues and annotation of genes encoding proteinaceous effectors in Lmb and Lml

The genome of Lmb encodes 13,047 proteins and that of Lml 11,272 proteins (Grandaubert et al. 2014; Dutreux et al. 2018). Orthologous proteins between Lmb and Lml were identified using OrthoFinder with default parameters (Emms and Kelly 2015).

Based on the new assembly and annotation available for the genome of Lmb (Dutreux et al. 2018), an updated repertoire of putative proteinaceous effector genes (encoding Small Secreted Proteins, SSP) has been predicted. Therefore, to compare effector repertoires of Lmb and Lml, the same pipeline for the prediction of putative effector genes was applied to both species. Signal peptide and subcellular localization were predicted by SignalP version 4.1 (Petersen et al. 2011) and TargetP version 1.1 (Almagro Armenteros et al. 2019) respectively. Transmembrane domains were predicted by TMHMM version 2.0. The predicted secretome contained all proteins with no more than one transmembrane domain and either a predicted signal peptide or a predicted extracellular localization. The final effector repertoires were created by applying a size cutoff of 300 amino acids on the predicted secretome. In parallel, EffectorP version 1.0 (Sperschneider et al. 2016) was used on the predicted secretome, and for Lml, four proteins were predicted as effectors with a size higher than 300 amino acids. These four proteins were added to the SSP set. This predicted a repertoire of 1080 and 892 SSP-encoding genes for Lmb and for Lml, respectively.

### Analysis of GO enrichment and statistical analyses

Gene Ontology (GO) annotations of the Lmb and Lml genes were retrieved from Dutreux et al. (2018) and Grandaubert et al. (2014) respectively. GO term enrichment analysis of the H3K4me2-, H3K9me3- and H3K27me3-associated genes was performed with the plug-in Biological networks Gene ontology (BinGo; v3.0.3) of the cytoscape software (Shannon et al. 2003). List of genes submitted to BINGO were considered as significantly enriched for a given GO term with an associated false discovery rate (FDR) ≤ 0.01 for the biological processes.

In order to assess significant enrichment of H3K4me2, H3K9me3 or H3K27me3 in certain categories of genes (such as proteinaceous or metabolic effector-encoding genes), a Chi^2^ test, or a Fisher’s exact test for small sized-population (Kim 2017), was applied to compare the expected and observed proportions of genes of a given category in H3K4me2, H3K9me3 or H3K27me3 domains (Soyer et al. 2019). Enrichment was considered significant with a *P* < 0.01. A Wilcoxon test was applied to identify significant differences in terms of the expression of genes associated with H3K4me2, H3K9me3 and H3K27me3 during axenic culture (considered significant with a *P* < 0.01). Analyses were done using R, version 3.0.2 (www.r-project.org).

## Results

### A different genome organization but similar epigenomic properties in both genomes

**Fig. 1:**
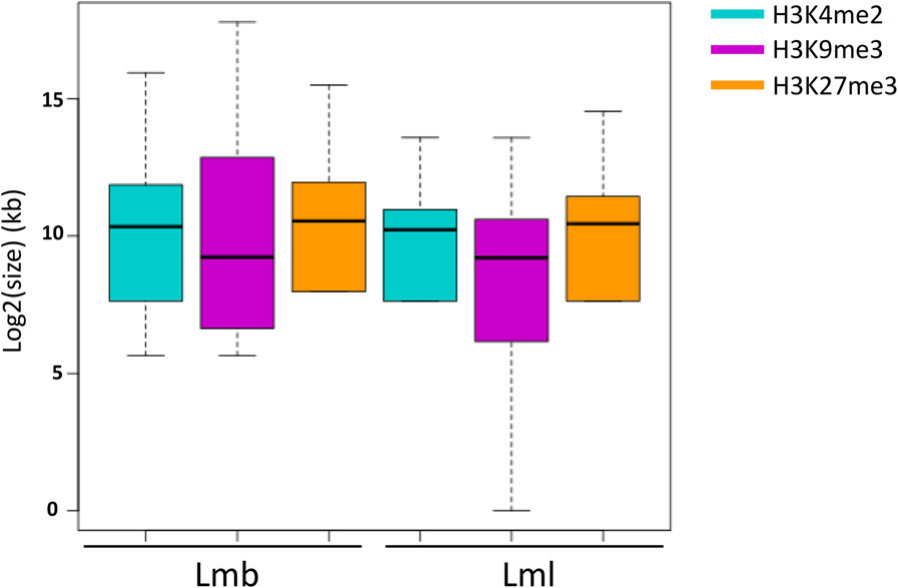
Size of the histone domains in the genomes of *Leptosphaeria maculans* ‘brassicae’ and *Leptosphaeria maculans* ‘lepidii’. Log2 of the size of the domains was estimated based on the coordinates of the location of the domains, identified using RSEG (Song and Smith 2011). Blue: H3K4me2; purple: H3K9me3; Orange: H3K27me3; Lmb, *L. maculans* ‘brassicae’; Lml, *L. maculans* ‘lepidii’

**Fig. 2:**
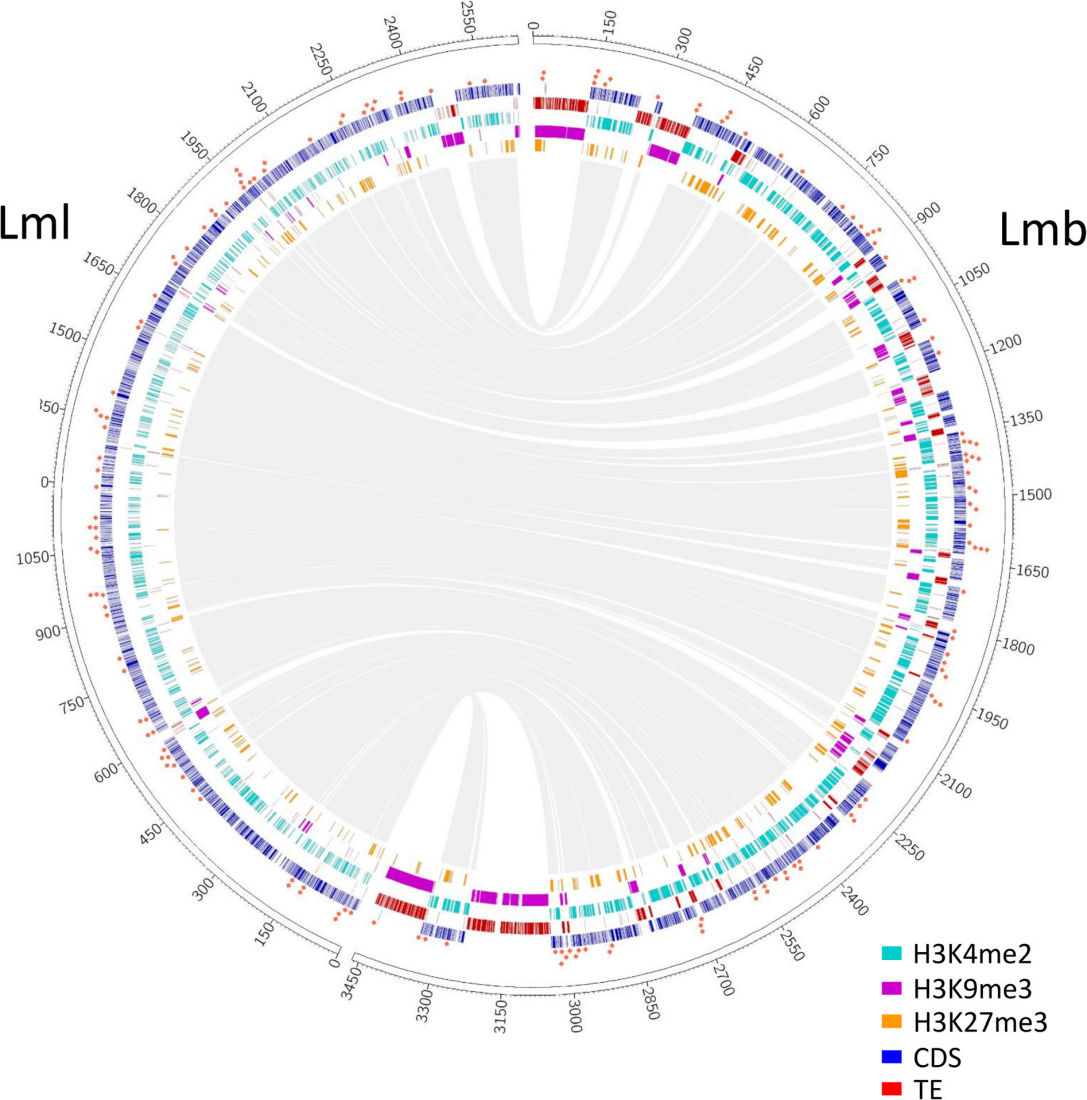
Genome of *Leptosphaeria maculans* ‘brassicae’ harbours large TE-rich, H3K9me3 domains compared to *Leptosphaeria maculans* ‘lepidii’. Example of SuperContig 2 of Lmb and Scaffold 1 of Lml. ChIP-seq was performed with antibodies targeting H3K4me2 (light blue), H3K9me3 (purple) or H3K27me3 (orange); rectangles indicate location of significantly enriched domains identified using RSEG (Song and Smith 2011). Location of CDS (dark blue); transposable elements (TE, red); H3K4me2 (light blue); H3K9me3 (purple); H3K27me3 (orange); Lmb, *L. maculans* ‘brassicae’; Lml, *L. maculans* ‘lepidii’. Genes encoding proteinaceous effectors are indicated with a red square

**Fig. 3:**
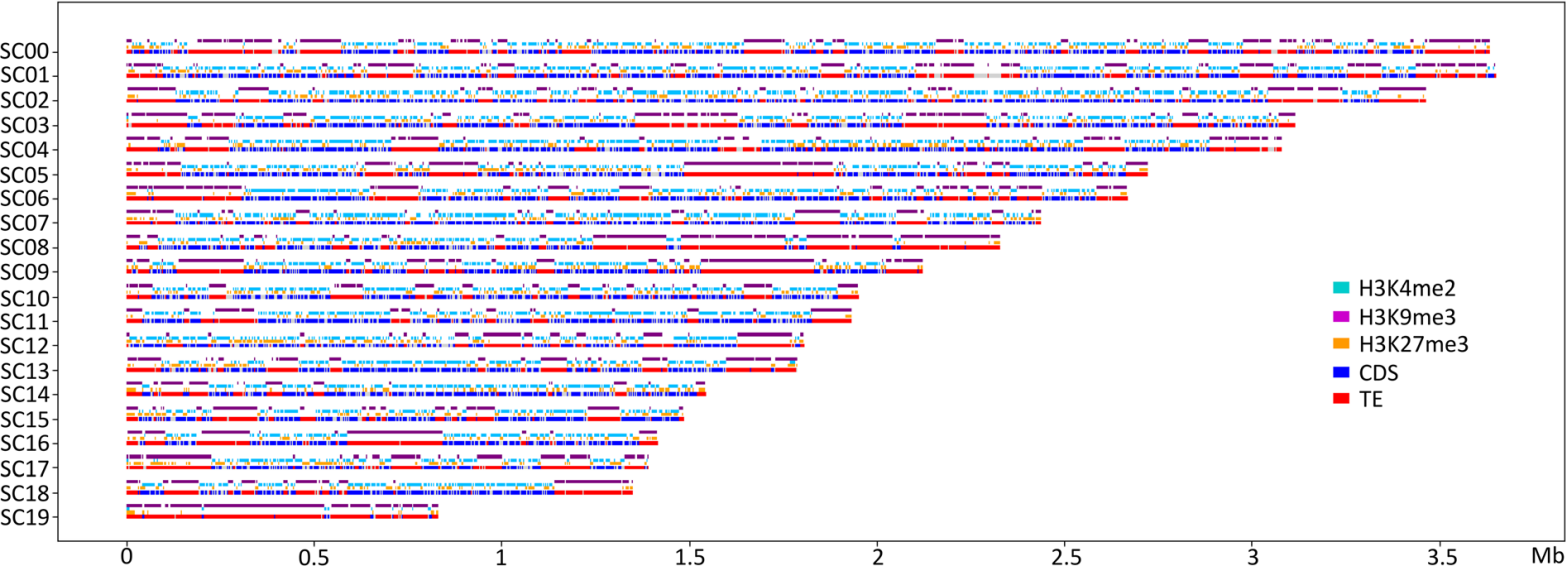
Location of different genome features in the genome of *Leptosphaeria maculans* ‘brassicae’. SuperContig (SC) 0 to 19 are displayed, representing 97% of the Lmb genome, with SC 19 being the dispensable chromosome. Location of CDS (dark blue); transposable elements (TE, red); H3K4me2 domains (light blue); H3K9me3 domains (purple); H3K27me3 domains (orange)

**Fig. 4:**
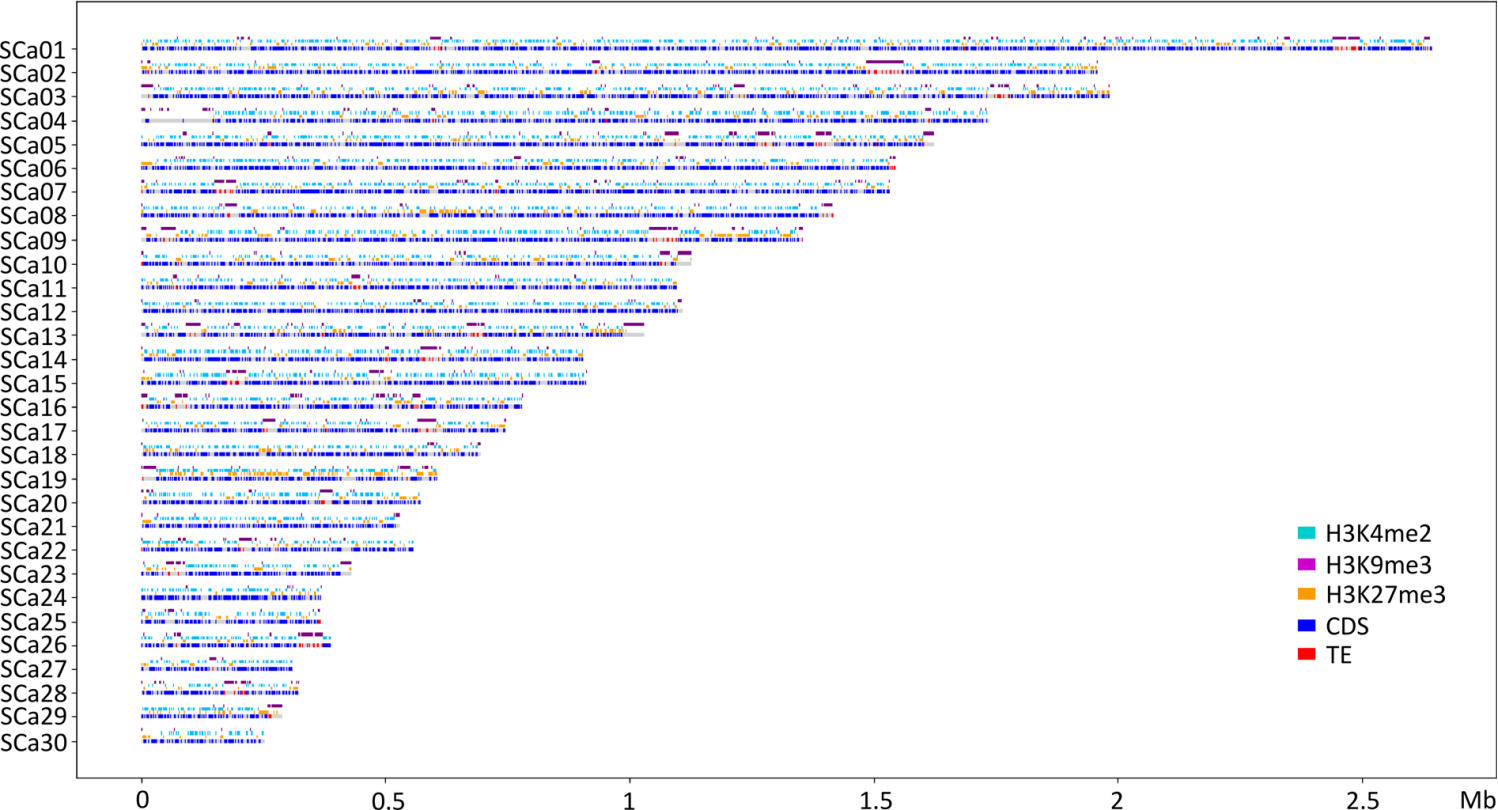
Location of different genome features in the genome of *Leptosphaeria maculans* ‘lepidii’. Scafflods (SCa) 1 to 30 are displayed, representing 93% of the Lml genome. Location of CDS (dark blue); transposable elements (TE, red); H3K4me2 domains (light blue); H3K9me3 domains (purple); H3K27me3 domains (orange)

To assess the genome-wide distribution of histone marks in Lmb and Lml during axenic growth, we performed ChIP-seq experiments using antibodies against histone modifications H3K4me2, H3K9me3 and H3K27me3 (Supplementary Table 1). Mapping of the ChIP-seq data was followed by identification of significantly enriched domains, for any of the histone modifications targeted, and reproducibility of the biological replicates was assessed (Supplementary Tables 2 and 3). Based on the genomic coordinates of the H3K4me2, H3K9me3 and H3K27me3 domains, the number of bases associated with any of the three histone modifications was evaluated, for each genome, in 10-kb sliding windows. In the genomes of Lmb and Lml, the proportion of H3K4me2 and H3K27me3 was similar (H3K4me2: 39% in Lmb vs. 32% in Lml; H3K27me3: 19% in Lmb vs. 13% in Lml) while the proportion of H3K9me3 was strikingly different (33% and 4% for Lmb and Lml, respectively) (Supplementary Tables 4 and 5). The average size of H3K4me2 and H3K9me3 domains were longer in Lmb than in Lml (H3K4me2: 2989 bp in Lmb vs. 1460 bp in Lml; H3K9me3 domains: 8443 bp in Lmb vs. 3499 bp in Lml). Notably, the Lmb genome displayed exceptionally large H3K9me3 domains, whereby one domain comprised up to 230 kb. In contrast, maximum size of H3K9me3 domains in Lml was only 12 kb. However, H3K27me3 domains were on average longer in the Lml than in the Lmb genome (1500 bp in Lmb vs. 2108 bp in Lml) (Figs. 1, 2, 3, and 4).

In the genomes of Lmb and Lml, H3K4me2 and H3K9me3 domains were mutually exclusive (Kendall’s *Ƭ*: −0.57 and −0.16, *P* < 2.2.10^−16^ for Lmb and Lml respectively; Figs. 2, 3, and 4; Supplementary Figure 1; Tables 1 and 2). We identified a positive correlation between the location of TEs and H3K9me3 domains (Kendall’s *Ƭ*: 0.87 and 0.68, *P* < 2.2.10^−16^ for Lmb and Lml respectively; Figs. 2, 3, and 4; Supplementary Figure 1; Tables 1 and 2) and between the location of coding sequences (CDS) and H3K4me2 (Kendall’s *Ƭ*: 0.55 and 0.25, *P* < 2.2.10^−16^ for Lmb and Lml respectively; Figs. 2, 3, and 4; Supplementary Figure 1; Tables 1 and 2). In the genome of Lmb, a weak correlation was demonstrated between CDS and H3K27me3 domains (Kendall’s *Ƭ*: 0.12, *P* < 2.2.10^−16^; Supplementary Figure 1; Table 1). For Lmb and Lml, the coverage of H3K9me3 and H3K27me3 was not homogeneous across all SCs because some are more than two fold enriched with these histone modifications compared to others (Supplementary Tables 4 and 5; Figs. 3 and 4). An extreme example of this pattern was identified on the dispensable chromosome of Lmb (i.e. SuperContig19; Leclair et al. 1996; Rouxel et al. 2011), which is strongly enriched in TEs compared to the rest of the genome (35% of TEs in the core genome and 93% of TEs in the dispensable chromosome). Consistently, we found a strong enrichment in H3K9me3 on the dispensable chromosome (32% in the core genome and 90% in the dispensable chromosome; Supplementary Table 4; Fig. 3), with only a few H3K4me2 and H3K27me3 domains (Fig. 3). In Lmb, we found that many sub-telomeric regions showed overlaps between H3K9me3 and H3K27me3, whereas these marks did not overlap on the chromosome arms, even within large TE-rich regions (Fig. 3). The assembly quality of the Lml genome however did not allow us to evaluate whether this was a common feature between the two species.

**Table 1:** Correlation between transposable elements, coding sequences and histone modifications in the *Leptosphaeria maculans* ‘brassicae’ genome

	CDS	TE	H3K4me2	H3K9me3	H3K27me3
CDS	1.00	−0.63	0.55	−0.60	0.12
TE	−0.63	1.00	−0.58	0.87	−0.25
H3K4me2	0.55	−0.58	1.00	−0.57	−0.11
H3K9me3	−0.60	0.87	−0.57	1.00	−0.24
H3K27me3	0.12	-0.25	−0.11	−0.24	1.00

The coverage of transposable elements (TE), coding sequences (CDS) and histone modifications (H3K9me3, H3K27me3, H3K4me2) along the genome of *L. maculans* ‘brassicae’ was analyzed in 1000-bp sliding windows. A Kendall’s *Ƭ* correlation analysis was done using R

**Table 2:** Correlation between transposable elements, coding sequences and histone modifications in the *Leptosphaeria maculans* ‘lepidii’ genome

	CDS	TE	H3K4me2	H3K9me3	H3K27me3
CDS	1.00	−0.22	0.25	−0.25	−0.05
TE	−0.22	1.00	−0.16	0.68	0.00
H3K4me2	0.25	−0.16	1.00	−0.16	−0.23
H3K9me3	−0.25	0.68	-0.16	1.00	0.01
H3K27me3	−0.05	0.00	−0.23	0.01	1.00

The coverage of transposable elements (TE), coding sequences (CDS) and histone modifications (H3K9me3, H3K27me3, H3K4me2) along the genome of *L. maculans* ‘lepidii’ was analyzed in 1000-bp sliding windows. A Kendall’s *Ƭ* correlation analysis was done using R

### H3K4me2 domains associate with genes involved in primary metabolism while H3K27me3 domains shelter genes involved in niche adaptation and cell wall degradation

**Fig. 5:**
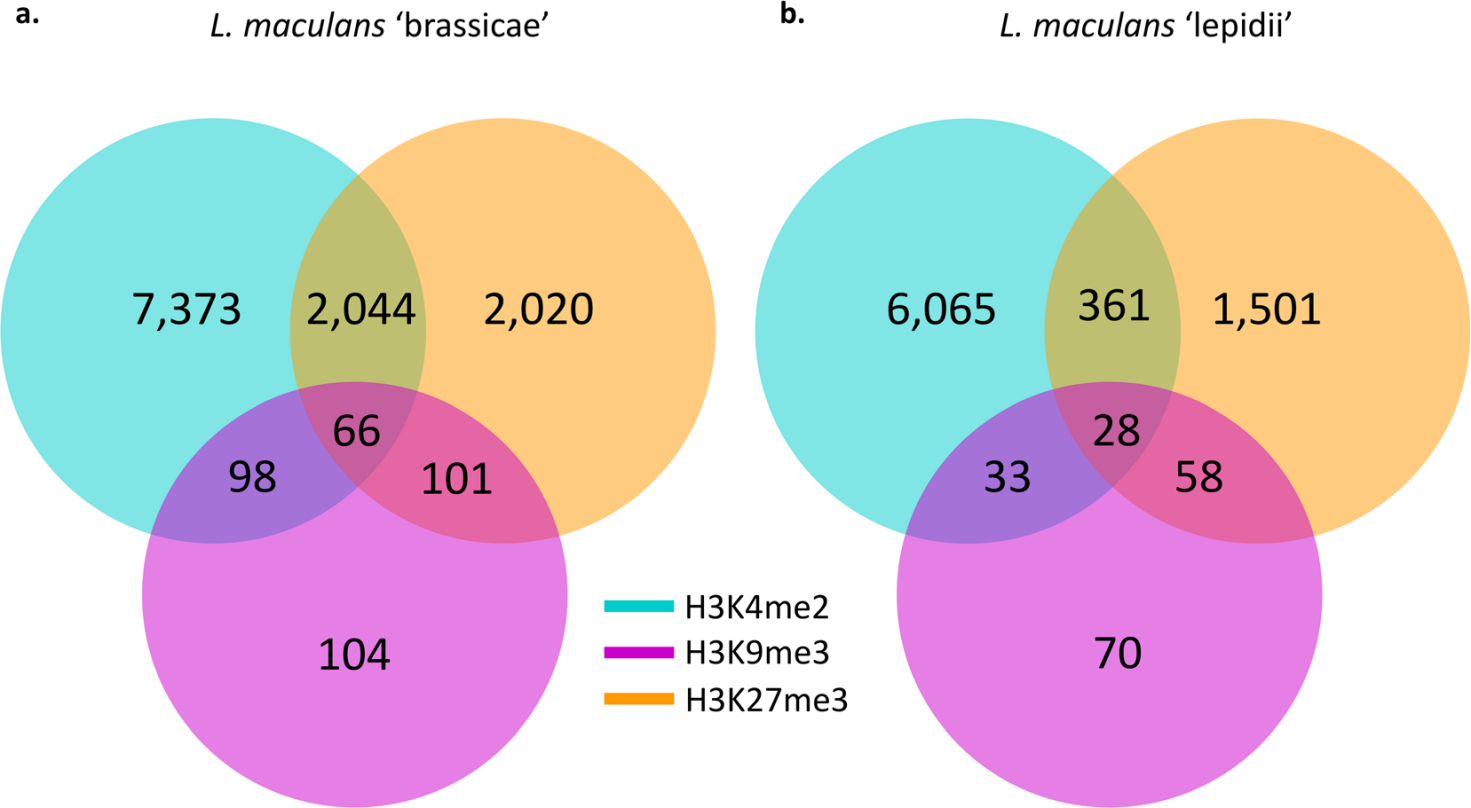
Number of genes associated with histone modifications in *Leptosphaeria maculans* ‘brassicae’ and *Leptosphaeria maculans* ‘lepidii’. (**a**) *L. maculans* ‘brassicae’, Lmb and (**b**) *L. maculans* ‘lepidii’, Lml. Locations of histone modifications in the genomes of Lmb and Lml were identified using RSEG (Song and Smith 2011). Blue: H3K4me2; purple: H3K9me3; orange: H3K27me3. Genes were considered as associated with any of the histone modifications when at least 1 bp of the gene was found within the borders of the domain

Genes associated with H3K4me2, H3K9me3 or H3K27me3 domains were identified in the genomes of Lmb and Lml during axenic growth. Overall, the number of genes associated with any of the histone modifications was similar in both species, with more than 50% of the predicted genes associated with H3K4me2, ~14% of the genes associated with H3K27me3 and only a few genes associated with H3K9me3 (104 and 70 respectively in Lmb and Lml; Fig. 5). In Lmb, there was a higher number of genes associated with both H3K4me2 and H3K27me3 than in Lml (2044 and 361 for Lmb and Lml, respectively) (Fig. 5). For both genomes, we could assign 30–40% of the predicted genes to a GO category (5076 and 3725 genes for Lmb and Lml, respectively; Grandaubert et al. 2014; Dutreux et al. 2018). We found that H3K4me2 was enriched with genes with a GO annotation (*X*^2^ test, *P* < 2.2.10^−16^) because more than 40% of these genes had a GO annotation (3276 and 2581 genes for Lmb and Lml respectively). Between 20 and 28% of the genes associated with H3K27me3 (572 and 288 genes for Lmb and Lml, respectively) had a GO annotation. However, none of the genes located in H3K9me3 domains, H3K9/K27me3 domains or H3K4me2/H3K27me3 domains had a GO annotation. In other words, genes located in heterochromatin were enriched with genes without a known function (*X*^2^ test, *P* < 2.2.10^−16^). For both species, genes associated with H3K4me2 displayed a wide variety of annotated functions corresponding to primary metabolism and basic cellular functions, such as translation (GO:0006412; 206 genes) or cellular protein metabolic process (Supplementary Tables 6, 7). For both species, only a few GO terms were identified among genes associated with H3K27me3. Nevertheless, in Lmb, these genes were significantly enriched in GO terms associated with carbohydrate metabolic process (GO:0005975; 82 genes), oxydo-reduction process (GO:0055114; 149 genes) and transmembrane transport (GO:0055085; 104 genes) (*P* ≤ 0.01) (Supplementary Table 8). As for Lml, probably due to the lower number of genes associated to H3K27me3, only a few GO annotations were detected. We only identified one GO category that was found in common with Lmb, namely carbohydrate metabolic process (GO:0005975; 41 genes). Other enrichments corresponded to a few genes classified as response to chemical (GO: 42221; 20 genes), response to nitrogen compound (GO:1901698; 10 genes), response to organophosphorus (GO: 46683, 10 genes) and others (Supplementary Table 9). Although most GO terms enriched among genes associated with H3K27me3 in Lmb and Lml did not overlap, GO terms identified suggest that H3K27me3-associated genes might not only be involved in stress response mechanisms, but may also be involved in feeding or cell wall degradation processes during plant infection.

### H3K4me2 domains are associated with expressed genes while H3K9me3 and H3K27me3 domains are associated with silent genes during axenic growth

**Fig. 6:**
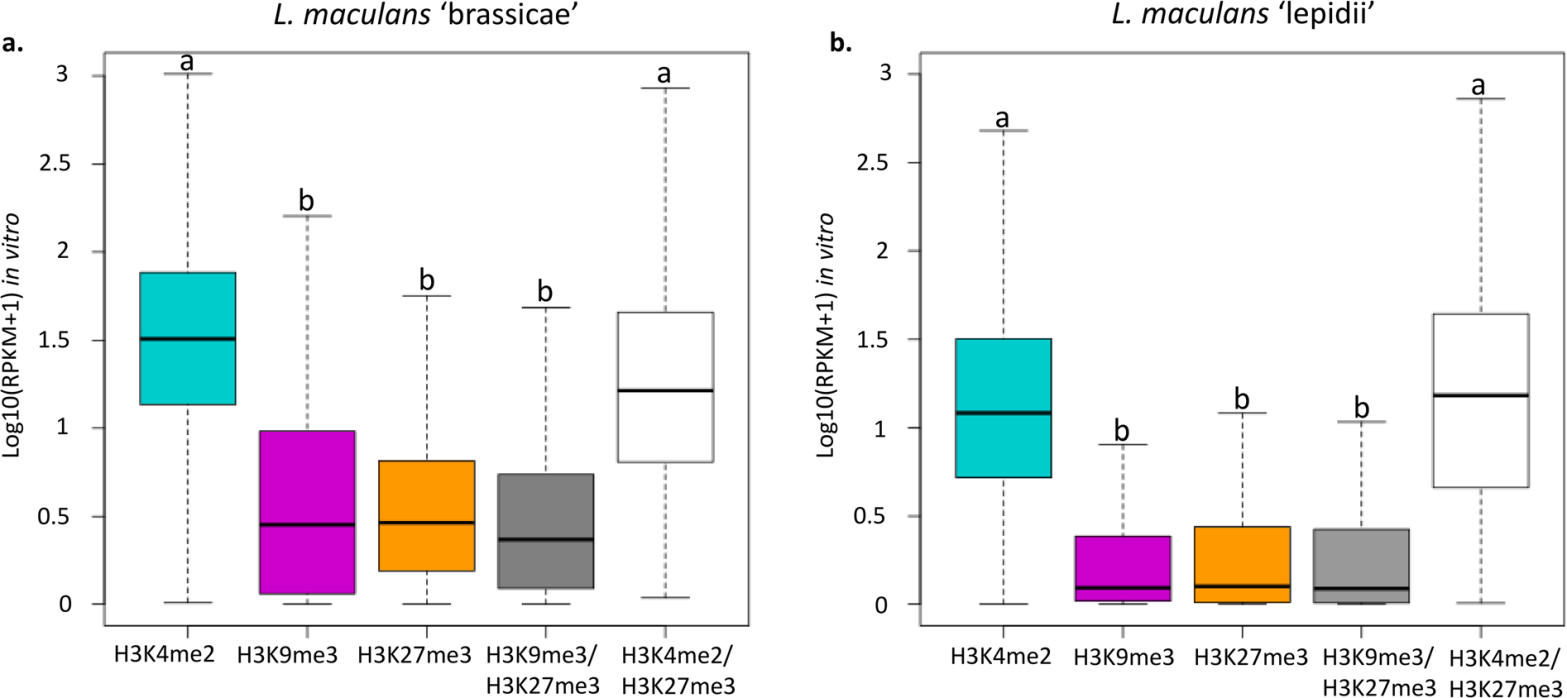
Genes associated with heterochromatin are less expressed than genes associated with euchromatin in *Leptosphaeria maculans* ‘brassicae’ and *Leptosphaeria maculans* ‘lepidii’. (**a**) *L. maculans* ‘brassicae’, Lmb; (**b**) *L. maculans* ‘lepidii’, Lml. Location of histone modifications in the genomes of Lmb and Lml was identified using RSEG (Song and Smith 2011), during axenic culture. RNA-seq was performed from Lmb or Lml grown one week in FRIES media. Blue: H3K4me2; purple: H3K9me3; orange: H3K27me3; grey: H3K9me3/H3K27me3; white: H3K4me2/H3K27me3

We performed a genome-wide transcriptome profiling of fungal cultures grown in vitro, and we correlated gene expression patterns with the distribution of histone modifications. In Lmb, 10,934 genes (83% of the predicted genes) and 7735 genes in Lml (69% of the predicted genes) were expressed during vegetative growth. Considering the top 100 most expressed genes of Lmb, 68 were associated with H3K4me2 while two were located within a H3K27me3 domain and none in a H3K9me3 domain (data not shown). In Lml, 71 were located within a H3K4me2 domain while five were located in a H3K27me3 domain. These data were confirmed at a genome-wide scale because H3K4me2 domains were enriched with genes expressed during axenic culture (7104 of the genes associated with H3K4me2 were expressed in Lmb and 5338 in Lml; Fig. 6; *P* < 2.2.10^−16^). On the contrary, H3K9me3 and H3K27me3 domains were enriched with genes that were silent during in vitro growth (Fig. 6; *P* < 2.2.10^−16^). On the other hand, in both species, genes that were associated with both H3K4me2 and H3K27me3 were expressed during axenic growth (90% and 87% of the genes, respectively, in Lmb and Lml) and their level of expression was similar to that of genes located in H3K4me2 domains (Fig. 6). We also investigated the influence of H3K4me2, H3K9me3 or H3K27me3 on expression of genes encoding proteinaceous effectors and observed that these histone modifications have the same effect on expression of effector genes as for any other genes (Supplementary Figure 2). We further assessed whether the extent to which a gene is associated with H3K4me2, H3K9me3 or H3K27me3 could influence the expression of the associated gene. We classified the genes in different categories based on their association with a particular histone modification and assessed whether this was correlated to the pattern of gene expression (Supplementary Figure 3). Overall, considering different types of coverage (either in the beginning or the end of the CDS, or a coverage of the entire gene), we found that H3K4me2 was associated with transcriptional activity while H3K9me3 and H3K27me3 were associated with transcriptional repression (Supplementary Figure 3; *P* < 0.01). In Lmb, genes entirely associated with H3K4me2 showed the highest level of expression while genes entirely associated with H3K9me3 or H3K27me3 showed less expression. Genes associated with H3K9me3 and H3K27me3 grouped together in terms of expression (Supplementary Figure 3; *P* < 0.01). In Lml, genes partially associated with H3K4me2 (either on the beginning or the end of the CDS) were more expressed than genes entirely associated with H3K4me2 (Supplementary Figure 3; *P* < 0.01). Contrary to Lmb, the less expressed genes of Lml were those associated with H3K27me3 (Supplementary Figure 3; *P* < 0.01), which might reflect that a very little amount of H3K9me3 is present in the genome of Lml. Thus, our analyses provide evidence that H3K27me3 might be the most important regulator of gene expression in the Lml genome. In both species, this transcriptomic analysis confirmed that genes located in a H3K4me2 domain were more likely to be expressed while those located in H3K9me3 and H3K27me3 domains were more likely to be silent.

### Heterochromatin domains are enriched with species-specific genes

A total of 7393 genes were conserved between both species. H3K4me2 domains were enriched with genes conserved between these two species, for example, 4892 Lmb genes located in euchromatin regions during growth in vitro were conserved (*X*^2^ test, *P* < 2.2.10^−16^) and the vast majority of these (4298 i.e. 88%) were expressed during axenic growth in both Lmb and Lml. Most of the conserved genes associated with H3K4me2 were involved in primary metabolism as mentioned above. Genes associated with both H3K4me2 and H3K27me3 were also enriched with conserved genes (1318 and 262 genes, respectively, in Lmb and Lml) representing more than 65% of the genes in these domains (*X*^2^ test, *P* < 5.2.10^−3^). In Lmb, two genes involved in heterochromatin assembly and maintenance were analyzed previously through gene silencing (*LmHP1* and *LmKMT1*/*DIM5*; Soyer et al. 2014). *LmHP1* is conserved in Lml and located in euchromatin in both species. *LmKMT1* is also conserved in both species, being located in a H3K4me2/H3K27me3 domain in Lmb and within a H3K4me2/H3K9me3 domain in Lml, during axenic growth. In contrast, H3K27me3 and H3K9me3 domains were significantly enriched with species-specific genes (a total of 865 genes and 661 genes, respectively, in Lmb and Lml; *X*^2^ test, *P* < 2.2.10^−16^). Among genes located in heterochromatin in both species (either H3K9me3, H3K27me3 or H3K9/K27me3 domains), 445 were conserved between Lmb and Lml, of which 83 were expressed and 182 were repressed during axenic growth in both species. Furthermore, among the orthologous genes, the genes associated with euchromatin in Lmb were also associated with euchromatin in Lml (as 83% of the genes conserved between the two species located in euchromatin in Lmb were also associated with euchromatin in Lml; Supplementary Table 10); half of the genes conserved between the two species and associated with H3K27me3 were also associated with H3K27me3 in Lml (Supplementary Table 10). On the contrary, conserved genes associated with heterochromatin in Lmb (either associated with H3K9me3 or H3K9me3/H3K27me3) were mostly found to be associated with H3K27me3 in the Lml genome (48% of the genes conserved between the two species associated with H3K9me3 or H3K9me3/H3K27me3 were found associated with H3K27me3 in the Lml genome; Supplementary Table 10). No GO enrichment was found for these genes and predicted functions were sparse, with less than 45% of them having a functional annotation. Strikingly, the main enrichment was in genes encoding putative effectors (16% of the genes).

### Heterochromatin domains are enriched with proteinaceous and metabolic effector genes

We then focused on candidate proteinaceous or metabolic effectors and wondered whether they were conserved and showed a distinct pattern of histone modifications. In the genome of Lmb, 2466 genes (i.e. 19% of the total genes) were associated with TE-rich regions (i.e. located within 2-kb distance of a TE sequence; Ohm et al. 2012), of which 289 genes encoded putative proteinaceous effectors. Hence, although a new prediction of the effector repertoire was performed here, based on the new assembly of the Lmb genome, TE-rich regions were significantly enriched with proteinaceous effector genes, as was already shown by Rouxel et al. (2011; *X*^2^ test, *P* = 5.8.10^−10^). Similarly, both H3K9me3- and H3K27me3-associated genes were significantly enriched with putative proteinaceous effectors, as they represented, respectively, 36% and 14% of the genes associated with these histone modifications in vitro (*X*^2^ test, *P* < 2.2.10^−16^; Table 3; Supplementary Table 11). Likewise, in Lml, TE-rich regions, H3K9me3 and H3K27me3 domains were significantly enriched with putative proteinaceous effector genes while H3K4me2 domains were significantly depleted in such genes compared to the rest of the genome (Table 3; Supplementary Table 11). Among the 1080 putative proteinaceous effector genes predicted in Lmb (8.2% of the total predicted genes) and the 892 putative effector genes of Lml (7.9% of the total predicted genes), 274 were conserved between both species. Hence, more than two-thirds of the effector repertoire is species specific (*X*^2^ test, *P* < 2.2.10^−16^), confirming our previous findings (Grandaubert et al. 2014). Overall, orthologous effector-encoding genes were associated with the same types of chromatin domains in Lmb and Lml. For example, 98% of the Lmb effector genes located in a euchromatin environment were also associated with H3K4me2 in Lml (82% of Lml effector genes located in euchromatin regions were also associated with H3K4me2 in Lmb). H3K27me3 and H3K9me3 domains were enriched with species-specific effector genes in both species (Table 3; Supplementary Table 11). As a striking example of the non-random location of effector genes in the two genomes, and the enrichment of heterochromatin regions with species-specific effectors, all currently known avirulence genes of Lmb were located in H3K9me3 domains in vitro, consistent with the fact that these genes are located in TE-rich genomic compartments; none of the nine was conserved in the genome of Lml. Twenty-eight other proteinaceous effector-encoding genes were located in TE-rich, H3K9me3 domains in Lmb, and only one of them had a putative ortholog in Lml. The 274 orthologous genes encoding effectors were then investigated for their distribution in euchromatic/heterochromatic regions during vegetative growth and for conservation of their location between orthologs. There was no obvious bias in the distribution of chromatin marks among the 274 genes, which is comparable to the overall distribution of marks among all genes of the effector repertoires (data not shown). At the individual gene level, 83 genes were likewise associated with H3K4me2 and 47 genes were likewise associated with H3K27me3 in both genomes.

**Table 3:** Number of proteinaceous effector genes located in different genomic compartments in *Leptosphaeria maculans* ‘brassicae’ and *Leptosphaeria maculans* ‘lepidii’

	*L. maculans* 'brassicae'								*L. maculans* 'lepidii'					
		Effector genes			Specific effector genes				Effector genes			Specific effector genes		
	Number of genes	Number	Proportion	*P* value^c^	Number	Proportion	*P* value^c^	Number of genes	Number	Proportion	*P* value^c^	Number	Proportion	*P* value^c^
Genome	13,047	1,080	8.2.10^-2^	-	806	6.2.10^-2^	-	11,272	892	7.9.10^-2^	-	618	5.5.10^-2^	-
TE-associated genes^a^	2,466	289	1.2.10^-1^	5.6.10^-10^	210	8.5.10^-2^	1.4.10^-6^	641	79	1.2.10^-1^	3.7.10^-8^	66	1.10^-1^	3.4.10^-11^
H3K4me2 domains^b^	7,373	433	5.8.10^-2^	6.7.10^-14^	319	4.3.10^-2^	4.1.10^-11^	6,065	266	4.4.10^-2^	3.9.10^-14^	163	2.7.10^-2^	5.8.10^-14^
H3K9me3 domains^b^	104	38	3.6.10^-1^	2.2.10^-16^	35	3.4.10^-1^	2.2.10^-16^	70	14	2.10^-1^	1.3.10^-5^	14	2.10^-1^	1.8.10^-9^
H3K27me3 domains^b^	2,020	286	1.4.10^-1^	2.2.10^-16^	200	9.9.10^-2^	3.6.10^-12^	1501	217	1.45.10^-1^	2.2.10^-16^	152	1.10^-1^	2.2.10^-16^
H3K9 + H3K27 me3 domains^b^	101	24	2.4.10^-1^	1.6.10^-8^	16	1.6.10^-1^	5.5.10^-5^	58	18	3.1.10^-1^	2.8.10^-13^	14	2.4.10^-1^	2.2.10^-16^

^a^Genes located up to 2 kb upstream or downstream of a transposable element sequence

^b^Genes located in a H3K4me2, H3K9me3, H3K27me3 or H3K9me3/H3K27me3 domain *in vitro*

^c^A *X*^2^ test was applied to compare proportion of effector genes in the genome and in the genomic compartment analysed

The genomes of Lmb and Lml contain secondary metabolite gene clusters including key genes encoding PKS (polyketide synthases) and NRPS (non-ribosomal peptide synthetases). Twenty-seven such genes were predicted in Lmb among which 24 were conserved in Lml, but they were overall absent from other closely related species (Rouxel et al. 2011; Grandaubert et al. 2014; Supplementary Table 12). Of these, only three have been experimentally demonstrated to be involved in Lmb pathogenicity, namely the PKS responsible for synthesis of abscisic acid (ABA), only expressed during cotyledon infection (Darma et al. 2019), the PKS responsible for producing phomenoic acid (Elliott et al. 2013) and the NRPS responsible for synthetizing sirodesmin, a toxin produced during stem infection (Gardiner et al. 2004; Elliott et al. 2007). The ABA PKS and all seven genes of the cluster, which are intermingled with three TE-rich regions, were entirely absent from the genome of Lml, while the other two were conserved (Supplementary Table 12). In Lmb, 81% of the PKS/NRPS-encoding genes were associated to H3K27me3 or H3K4me2/H3K27me3 domains (22 PKS/NRPS; Fisher’s exact test, *P* = 1.3.10^−7^). Likewise in the Lml genome, H3K27me3 domains were enriched with PKS/NRPS-encoding genes (12 PKS/NRPS; Fisher’s exact test, *P* = 1.7.10^−4^), and all of them had orthologs associated with similar marks in Lmb. The similar chromatin context also resulted in similar regulation in most of the cases, with 19 of the orthologs being similarly expressed during in vitro growth (17 expressed and two repressed; Supplementary Table 12).

## Discussion

The sister species *L. maculans* ‘brassicae’ and *L. maculans* ‘lepidii’ exhibit marked differences in their genome organization. Notably, Lmb has large TE-rich domains structuring the genome into alternating gene-rich and TE-rich regions (Rouxel et al. 2011). In the genome of Lml, in contrast, TEs are evenly distributed across the genome and no compartmentalization of the genome is evident in relation to TE location (Grandaubert et al. 2014). The massive invasion of the Lmb genome by TEs occurred ca. 5 MYA and was postulated to have been instrumental in the separation of the two species (Grandaubert et al. 2014). This invasion may also have contributed to the rise of Lmb as a successful pathogen of *B. napus* due to the specific localization of a number of candidate effector genes in TE-rich regions of the genome (Rouxel et al. 2011; Grandaubert et al. 2014). Differences in genomic organization between these two closely related species could impact the underlying epigenomic landscape and have important consequences for fungal biology and pathogenicity. This could provide distinct strategies to regulate the expression of genes involved in stress response or pathogenicity, as it was shown in Lmb that histone modification H3K9me3 is involved in the repression of avirulence genes during axenic growth (Soyer et al. 2014). To investigate whether different genomic organization impact the underlying epigenomic organization, we here compared the genome-wide location of three different histone modifications, typically associated with euchromatin or heterochromatin, in these two closely related phytopathogenic fungi, during axenic culture. We found that differences in the epigenomic landscape of Lmb and Lml are in accordance with their genome organization. In Lmb, very large H3K9me3 domains are present, spanning large TE-rich regions, while such extremely large H3K9me3 domains are not observed in the Lml genome. Our findings corroborate previous epigenetic analyses of a few genes performed in vitro, pointing out that avirulence genes are located in heterochromatin (Soyer et al. 2014), but also demonstrate that heterochromatin (either associated with H3K9me3 or H3K27me3) is enriched with putative effector genes (Soyer et al. 2019).

Although Lmb and Lml have distinct genome organizations, they share a common distribution of histone modifications throughout their genome during axenic culture. Gene-rich regions are enriched with H3K4me2 and H3K27me3, while TE-rich regions are associated with H3K9me3. The proportion of H3K9me3 in a genome often reflects the TE content, as was described in *Z. tritici* or *Podospora anserina* (Schotanus et al. 2015; Carlier et al. 2020). In Lmb, having more than 30% of TE, 33% of the genome is associated with H3K9me3 while the genome of Lml, having a low TE content, shows a low enrichment in H3K9me3 (4% of H3K9me3). Domains enriched with H3K4me2 and domains enriched with H3K9me3 are mutually exclusive in the genomes of Lmb and Lml, as was shown in *Neurospora crassa*, *Fusarium fujikuroi*, *Z. tritici* or *P. anserina* (Smith et al. 2008; Jamieson et al. 2013; Wiemann et al. 2013; Schotanus et al. 2015; Carlier et al. 2020). H3K27me3 has been detected in most filamentous fungi investigated so far, except in *Mucor*, *Rhizopus* or Aspergilli such as *Aspergillus nidulans* (Gacek-Matthews et al. 2016; reviewed in Freitag 2017). In sub-telomeric regions of Lmb, H3K9me3 and H3K27me3 domains overlap over repetitive sequences, which is also the case in *N. crassa* or *Z. tritici* (Smith et al. 2008; Jamieson et al. 2013; Jamieson et al. 2018; Schotanus et al. 2015). In the genome of Lmb, TE-rich regions are enriched with H3K9me3 but, except for the sub-telomeric regions, no enrichment in H3K27me3 was associated with TEs on chromosomal arms. This contrasts with the organization of TE-rich regions in *Z. tritici*, which are enriched with both heterochromatin modifications (Schotanus et al. 2015). The situation in Lmb is also different from that of *Fusarium oxysporum* in which most TE sequences are embedded in H3K27me3 domains (Fokkens et al. 2018). We confirmed that, as in most eukaryotes, H3K4me2 domains are associated with expressed genes while H3K9me3 and H3K27me3 domains are associated with silent genes. In Lmb, although the locations of H3K4me2 and H3K27me3 domains do not overlap at a genome-wide scale, more than 2000 genes were found to be associated with both histone methylations. This is strikingly different to Lml, where only 300 genes were associated with both histone modifications, and *Z. tritici*, where 400 genes are embedded in such domains (Schotanus et al. 2015; Soyer et al. 2019). Bivalent domains are defined as chromatin regions associated with both repressive and permissive histone modifications (Bernstein et al. 2006). The large number of genes associated with H3K27me3 and H3K4me2 during axenic culture in Lmb might indicate a biological specificity of this species and the existence of bivalent domains, because these two modifications have antagonistic effects on gene expression (Harikumar and Meshorer 2015). Altogether, our findings show that, despite differences in genomic organization between Lmb and Lml (and the other fungi in which epigenomic analyses were so far performed), the epigenomic landscape is overall conserved.

While H3K9me3 and H3K27me3 are signatures of heterochromatin, H3K9me3 is considered to be typical of constitutive heterochromatin, being associated with repeats and involved in genome stability, whereas H3K27me3 is considered to be associated with facultative heterochromatin that is easily reversed to a euchromatin state under certain abiotic and biotic stress conditions (Grewal and Jia 2007). However, dogmas regarding conventional definitions of facultative or constitutive heterochromatin seem to be challenged in fungi, or at least in the few plant pathogenic fungi in which epigenomic analyses have been performed. While the co-localization of H3K9me3 and TEs in the Lmb and Lml genomes is consistent with a constitutive heterochromatin state, the fact that H3K9me3 domains encompass genes that are expressed during interaction with oilseed rape suggests that these regions may correspond to a category of facultative heterochromatin in Lmb. Genes associated with H3K9me3 are almost all located in the middle of repeated elements, in sub-telomeric areas, or very close to the edge of regions enriched with repeated elements. Moreover, some of these genes, including the nine cloned avirulence genes of Lmb which are located within AT-rich isochores (e.g. *AvrLm1* or *AvLm6*; Gout et al. 2006; Fudal et al. 2007) including sub-telomeric regions (e.g*. AvrLm3* or *AvrLm10*; Plissonneau et al. 2016; Petit-Houdenot et al. 2019), are highly transcribed upon host infection (Rouxel et al. 2011). This finding, together with other studies, questions the ‘constitutive’ nature of heterochromatin associated with H3K9me3. In contrast, the location of H3K27me3 in the genomes of Lmb and Lml supports its association with facultative heterochromatin. Indeed, we found H3K27me3 associated with coding sequences and enriched with genes encoding proteinaceous and metabolic effectors or proteins involved in stress responses. Most of these genes are silenced during vegetative growth but induced during plant infection. In fungi, recent analyses also highlighted a role for H3K27me3 in genome organization and stability. For instance, the dispensable chromosomes of *Z. tritici* are twice as rich in TEs as the core chromosomes, and whereas there is no significant enrichment in H3K9me3 on the dispensable chromosomes, they are entirely covered by H3K27me3 (Schotanus et al. 2015). In *Z. tritici*, the loss of H3K27me3 was found to increase the stability of some accessory chromosomes (Möller et al. 2019). In *Z. tritici* and *N. crassa*, H3K27me3 is relocated towards normal constitutive heterochromatin (i.e. H3K9me3 domains) under genotoxic stress, such as the loss of H3K9me3 after inactivation of *KMT1* (Basenko et al. 2015; Möller et al. 2019). Altogether, these findings suggest that although H3K27me3 is an important regulator of gene expression involved in development or response to various stresses, it also plays a role in the maintenance of genome integrity. In Lmb and Lml, no particular association of H3K27me3 with TEs was identified and the dispensable chromosome of Lmb is not enriched with H3K27me3. The only association of H3K27me3 with constitutive chromatin was at the chromosome ends of Lmb, where we found overlaps between H3K9me3 and H3K27me3. Inactivation of *KMT1* and *KMT6* would help investigate whether H3K27me3 could also be involved in genome stability and relocation of heterochromatin marks in Lmb and Lml.

The heterochromatin domains of Lmb and Lml are rich in species-specific genes, genes involved in stress responses and putative proteinaceous or metabolic effectors. This supports the view that chromatin remodelling mechanisms are an efficient way to rapidly modulate gene expression under stress conditions or during biotic interactions, although the dynamics of chromatin structure might not be the sole regulator of these complex biological processes (Soyer et al. 2015a; Tan and Oliver 2017). This pattern is conserved in other plant-associated fungi, independently of their mode of interaction with their host (Ma et al. 2010; Rouxel et al. 2011; Chujo and Scott 2014; Schotanus et al. 2015; Faino et al. 2016; Vlaardingerbroek et al. 2016; Dallery et al. 2017; Krishnan et al. 2018; Soyer et al. 2019). Even outside of TE-rich regions, heterochromatin is often found enriched with pathogenicity-related genes, whether they encode proteinaceous effectors or are involved in the production of secondary metabolites (e.g. Connolly et al. 2013; Wiemann et al. 2013; Chujo and Scott 2014; Schotanus et al. 2015; Fokkens et al. 2018; Soyer et al. 2019). Importantly, sets of genes upregulated during host infection are found associated with heterochromatin in vitro (Fokkens et al. 2018; Haueisen et al. 2018; Soyer et al. 2019). One of our initial postulates was that invasion of the *L. maculans* genome by TEs contributed to the rise of Lmb as a pathogen specialized on Brassicas with greater pathogenic abilities than the non-invaded sister species. While Lml has never been found to be able to infect *B. napus* under our experimental conditions or isolated from our experimental fields (M-H. Balesdent & T. Rouxel, unpublished data), previous work reported its ability to infect other crucifers (Petrie 1969). Its isolation from ascocarps on stems of *Lepidium* sp. also suggests its infection strategy is similar to that of Lmb on *B. napus*. So far, all avirulence genes of Lmb, which can be recognized by the plant immune system to set up defence reactions, are located in TE-rich regions and associated with H3K9me3. In contrast, in both species, we found conserved putative effector genes (either proteinaceous or metabolic) harboured in similar H3K27me3 heterochromatin environments. In Lmb, some of them are expressed during stem infection of oilseed rape and have been named ‘late’ effector genes (Gervais et al. 2017). The contrasting heterochromatic environment (H3K9me3 vs. H3K27me3) for avirulence genes and ‘late’ effector genes might reflect a very different adaptive behaviour because effector genes that are expressed early, and likely to be ‘recognized’ by the plant surveillance machinery at the onset of penetration, are also those which are subject to accelerated evolution under selection (Rouxel et al. 2011). This suggests that there may be a selective advantage for the fungus to partition genes more likely to be recognized by the host plant within H3K9me3 domains. Nevertheless, the location of putative pathogenicity genes, including orthologues, in similar heterochromatin regions in the genomes of Lmb and Lml suggests that basic pathogenicity programs are independent of genome invasion by TEs, and points to the likewise importance of chromatin context on transcriptome shaping during infection. Taken together, these data support our previous hypothesis that the localization of effector genes in plastic genomic compartments is an efficient way to regulate the expression of sets of genes scattered throughout the genome that are involved in similar biological processes (Soyer et al. 2015a).

## Conclusions

To the best of our knowledge, previous comparative analyses of histone modifications have been performed in model organisms such as mouse (Mikkelsen et al. 2010) but none considered multiple histone modifications (typical for both heterochromatin and euchromatin) in closely related phytopathogenic fungi. So far, little is known about the extent of conservation of histone modifications among closely related species and how this may influence pathogenesis. Comparative genomics has allowed analyses of gene evolution (notably proteinaceous effectors), the location of effector genes in genomes or the diversity of effector repertoires in relation to host specialization or fungal lifestyles. The role of the epigenome is increasingly recognized in plant pathogenic fungi as an important regulator of genome structure (e.g. Basenko et al. 2015; Möller et al. 2019, 2020) and the expression of genes encoding effectors (e.g. Chujo and Scott 2014; Soyer et al. 2014, 2019; Meile et al. 2020). The next step will be to exploit epigenomic data in a comparative framework to better understand the role of the epigenomic landscape in adaptation of pathogens to environmental changes, modulation of interactions with the holobiont, host adaptation and specialization.

## Supplementary information

Supplementary Figure 1 **Correlation analysis of the location of genes, transposable elements and domains enriched for H3K4me2, H3K9me3 and H3K27me3 in the genomes of (a)** ***Leptosphaeria maculans*** **‘brassicae’; (b)** ***Leptosphaeria maculans*** **‘lepidii’.** Regions significantly enriched for any of the three histone modifications analyzed were identified through ChIP-seq performed during axenic culture; correlation analyses were performed using a Kendall’s *Ƭ* (see Materials and Methods). K4: H3K4me2; K9: H3K9me3; K27: H3K27me3; CDS: Coding sequences; TE: Transposable Elements. (PNG 5907 kb)

High Resolution (TIFF 1392 kb)

Supplementary Figure 2 **Influence of the association with H3K4me2, H3K9me3, H3K27me3 on effector gene expression during axenic culture.** (a) *L. maculans* ‘brassicae’, Lmb; (b) *L. maculans* ‘lepidii’, Lml. Location of histone modifications in the genomes of Lmb and Lml were identified using RSEG (Song and Smith 2011), during axenic culture. RNA-seq was performed from Lmb or Lml grown one week in FRIES media. Green: all genes encoding proteinaceous effectors; effectors associated with blue: H3K4me2; purple: H3K9me3; orange: H3K27me3; grey: H3K9me3+H3K27me3; white: H3K4me2+H3K27me3. (PNG 6596 kb)

High Resolution (TIF 1960 kb)

Supplementary Figure 3 **H3K4me2 is permissive for gene expression while H3K9me3 and H3K27me3 are restrictive for gene expression, regardless of the region of the gene associated with the histone modification.** (a) *L. maculans* ‘brassicae’, Lmb; (b) *L. maculans* ‘lepidii’, Lml. Location of histone modifications in the genomes of Lmb and Lml were identified using RSEG (Song and Smith 2011), during axenic culture. RNA-seq was performed from Lmb or Lml grown one week in FRIES media. Different shades of blue: H3K4me2; purple: H3K9me3; orange: H3K27me3. Genes associated with a given histone modification in the 5’: beginning of the CDS; 3’: end of the coding sequence; all: the entire coding sequence; over: partial association in other parts of the gene. (PNG 8794 kb)

High Resolution (TIF 2581 kb)

Supplementary Table 1 **Statistics of ChIP-seq and RNA-seq datasets and alignments.** Table shows the number of reads used for the alignment, the number of reads mapping only at one location, the number of reads aligned more than once and the unmapped reads against the genome of *L. maculans* 'brassicae' (Lmb; Dutreux et al. 2018) or *L. maculans* 'lepidii' (Lml; Grandaubert et al. 2014). (DOCX 20.6 kb)

Supplementary Table 2 **Correlation analysis between the different ChIP experiments generated with antibodies targeting histone modifications H3K4me2, H3K9me3 and H3K27me3 in** ***Leptosphaeria maculans*** **'brassicae', during** ***in vitro*** **growth.** Correlation analyses were performed to analyze location of the significantly enriched domains, identified using RSEG (Song and Smith 2011), from the three different biological replicates generated and analyzed separately. A Kendall's *Ƭ* correlation test was performed using R. (DOCX 20.5 kb)

Supplementary Table 3 **Correlation analysis between the different ChIP experiments generated with antibodies targeting histone modifications H3K4me2, H3K9me3 and H3K27me3 in *Leptosphaeria maculans* ‘lepidii’, during in vitro growth.** Correlation analyses were performed to analyse location of the significantly enriched domains, identified using RSEG (Song and Smith 2011), from the three different biological replicates generated and analyzed separately. A Kendall's *Ƭ* correlation test was performed using R. (DOCX 18 kb)

Supplementary Table 4 **Coverage of histone modifications H3K4me2, H3K9me3 and H3K27me3 in the genome of *Leptosphaeria maculans* ‘brassicae’.** ^a^Genome as published in Dutreux et al. (2018). ^b^Location of H3K4me2, H3K9me3 and H3K27me3 was determined through ChIP-seq analysis, in vitro, and regions significantly enriched for any of the modifications was identified using RSEG (Song and Smith 2011). (DOCX 19 kb)

Supplementary Table 5 **Coverage of histone modifications H3K4me2, H3K9me3 and H3K27me3 in the genome of *Leptosphaeria maculans* ‘lepidii’.** ^a^Genome as published in Grandaubert et al. (2014). ^b^Location of H3K4me2, H3K9me3 and H3K27me3 was determined through ChIP-seq analysis, in vitro, and regions significantly enriched for any of the modifications was identified using RSEG (Song and Smith 2011). (DOCX 26.8 kb)

Supplementary Table 6 **GO categories enriched in genes associated with H3K4me2 during axenic culture of** ***Leptosphaeria maculans*** **‘brassicae’.** GO annotation of the Lmb genes was retrieved from Dutreux et al. (2018). Analysis of GO enrichment among the genes associated with H3K4me2 during axenic culture of Lmb was performed using Cytoscape (Shannon et al. 2003). (DOCX 20.4 kb)

Supplementary Table 7 **GO categories enriched in genes associated with H3K4me2 during axenic culture of** ***Leptosphaeria maculans*** **'lepidii'.** GO annotation of the Lml genes was retrieved from Grandaubert et al. (2014). Analysis of GO enrichment among the genes associated with H3K4me2 during axenic culture of Lml was performed using Cytoscape (Shannon et al. 2003). (DOCX 18.9 kb)

Supplementary Table 8 **GO categories enriched in genes associated with H3K27me3 during axenic culture of** ***Leptosphaeria maculans*** **'brassicae'.** GO annotation of the Lmb genes was retrieved from Dutreux et al. (2018). Analysis of GO enrichment among the genes associated with H3K27me3 during axenic culture of Lmb was performed using Cytoscape (Shannon et al. 2003). (DOCX 17.2 kb)

Supplementary Table 9 **GO categories enriched in genes associated with H3K27me3 during axenic culture of** ***Leptosphaeria maculans*** **'lepidii'.** GO annotation of the Lml genes was retrieved from Grandaubert et al. (2014). Analysis of GO enrichment among the genes associated with H3K27me3 during axenic culture of Lml was performed using Cytoscape (Shannon et al. 2003). (DOCX 19.2 kb)

Supplementary Table 10 **Analysis of the localization of genes conserved between** *Leptosphaeria maculans* ‘brassicae’ and *Leptosphaeria maculans* **‘lepidii’, in relation to domains enriched with H3K4me2, H3K9me3, H3K27me3, H3K4me2/H3K27me3 and H3K9me3/H3K27me3 during axenic culture**. ^a^Number of genes located in a H3K4me2-, H3K9me3-, H3K27me3-, H3K4me2/H3K27me3-, H3K9me3/H3K27me3-domain in Lmb and conserved in Lml; ^b^location of the conserved genes of Lml in a H3K4me2-, H3K9me3-, H3K27me3-, H3K4me2/H3K27me3-, H3K9me3/H3K27me3-domain. (DOCX 16 kb)

Supplementary Table 11 **Analysis of the enrichment of effector- or specific effector-genes in TE-rich regions, H3K4me2-, H3K9me3-, H3K27me3-, H3K9me3/H3K27me3-domains in the genomes of *Leptosphaeria maculans* ‘brassicae’ or *Leptosphaeria maculans* ‘lepidii’.** ^a^Genes located up to 2 kb upstream or downstream of a transposable element sequence; ^b^Genes located in a H3K4me2-, H3K9me3-, H3K27me3- or H3K9me3/H3K27me3-domain in vitro; ^c^A *X*^*2*^ test was applied to compare proportion of effector genes in the genome and in the genomic compartment analyzed; ^d^Odds ratio was reported as a measure of the effect size. (DOCX 18 kb)

Supplementary Table 12 **Location of (putative) metabolic effector in ***Leptosphaeria maculans*** ‘brassicae’ and ***Leptosphaeria maculans*** ‘lepidii’ genomic compartments. **^a^Genes located up to 2 kb upstream or downstream of a transposable element sequence; ^b^Genes with RPKM≥ 2; ^c^Genes located in a H3K4me2-, H3K9me3-, H3K27me3- or H3K4me2/H3K27me3-domain *in vitro*; K4: H3K4me2; K9: H3K9me3; K27:H3K27me3. (DOCX 20.8 kb)

## Data Availability

Omic data are available under the GEO accession number GSE150127.

## Abbreviations

bp: base pair
CDS: coding sequence
ChIP-seq: chromatin immunoprecipitation followed by high-throughput sequencing
FDR: false discovery rate
GO: Gene Ontology
HP1: heterochromatin protein 1
Kb: kilobase
KMT: lysine methyltransferase
DIM: defective in methylation
H3K4me2: dimethylation of the lysine 4 of histone H3
H3K9me3: trimethylation of the lysine 9 of histone H3
H3K27me3: trimethylation of the lysine 27 of histone H3
Lmb: *Leptosphaeria maculans* ‘brassicae’
Lml: *Leptosphaeria maculans* ‘lepidii’
NRPS: non-ribosomal peptide synthetase
PKS: polyketide synthase
RPKM: reads per kilobase per million mapped reads
SC: SuperContig
SSP: small secreted protein
TE: transposable element
