# Supplementary figures and images for: Genome-wide mapping of histone modifications during axenic growth in two species of Leptosphaeria maculans showing contrasting genomic organization
Source: Chromosome Res. 2021 May 21;29(2):219–36. doi: 10.1007/s10577-021-09658-1 (PMC8159818; doi:10.1007/s10577-021-09658-1)

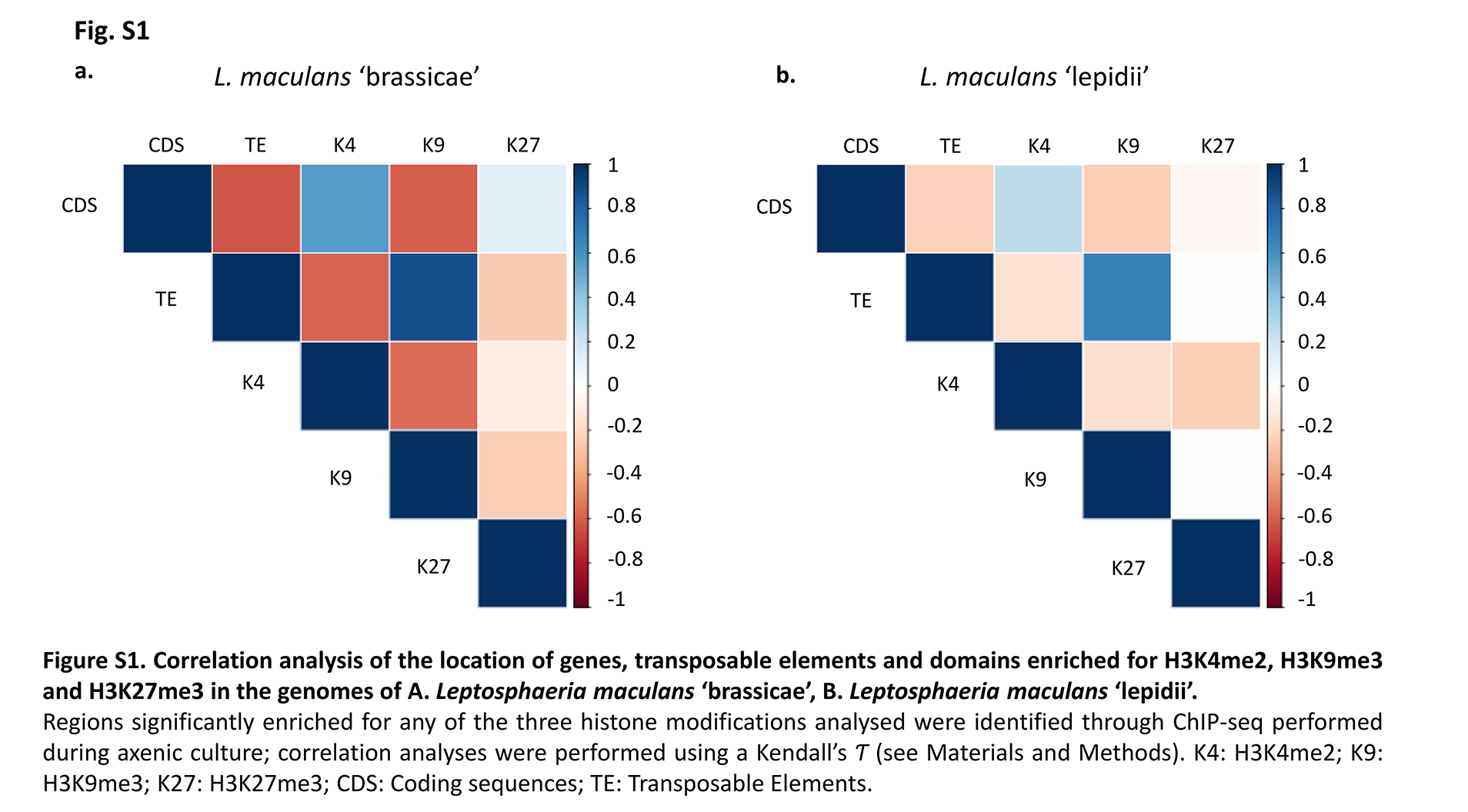

Supplement: Supplementary file 1 — Correlation analysis of the location of genes, transposable elements and domains enriched for H3K4me2, H3K9me3 and H3K27me3 in the genomes of (a) Leptosphaeria maculans ‘brassicae’; (b) Leptosphaeria maculans ‘lepidii’. Regions significantly enriched for any of the three histone modifications analyzed were identified through ChIP-seq performed during axenic culture; correlation analyses were performed using a Kendall’s Ƭ (see Materials and Methods). K4: H3K4me2; K9: H3K9me3; K27: H3K27me3; CDS: Coding sequences; TE: Transposable Elements. (PNG 5907 kb) [file 10577_2021_9658_Fig7_ESM.png]

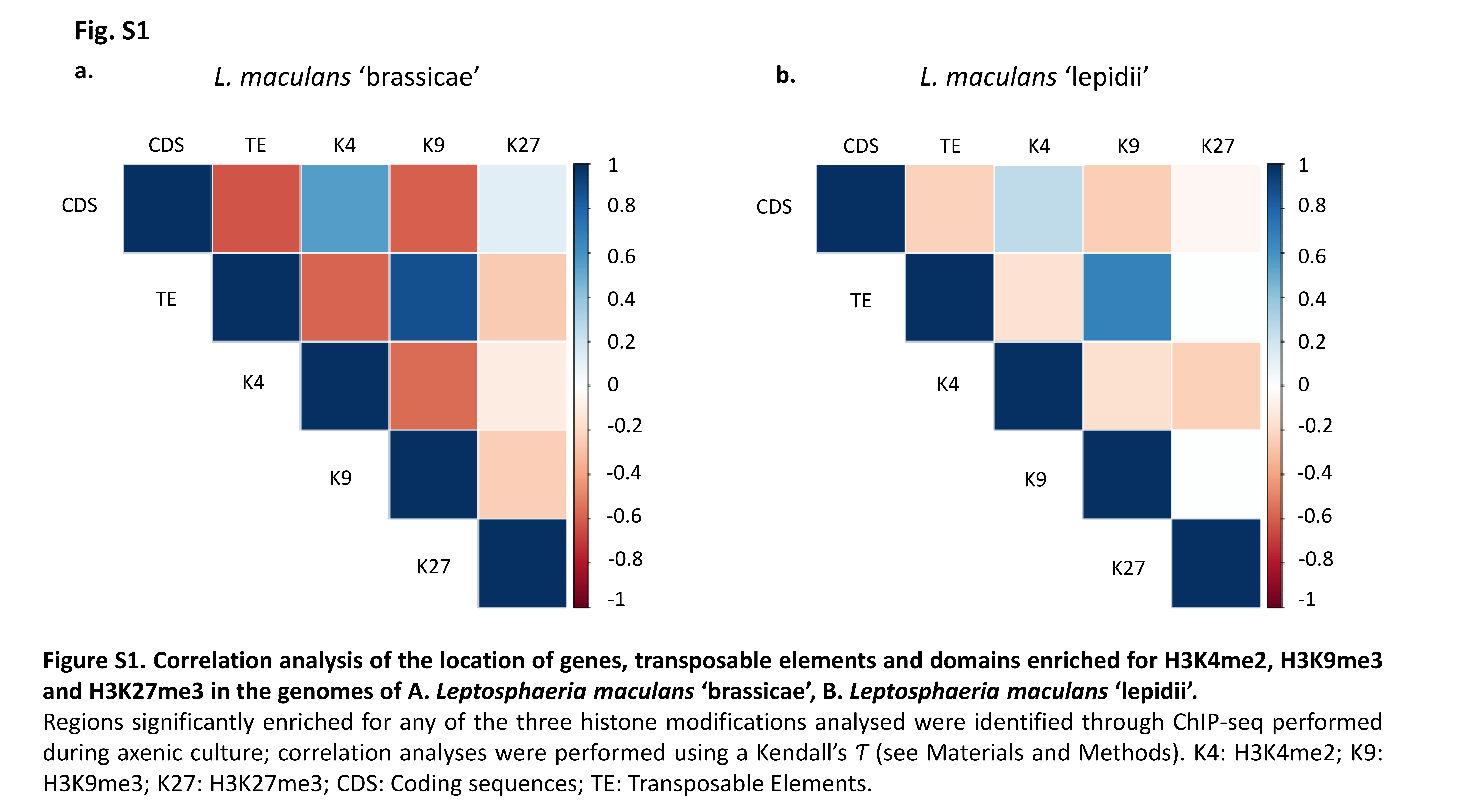

Supplement: Supplementary file 2 — High Resolution (TIFF 1392 kb) [file 10577_2021_9658_MOESM1_ESM.tiff]

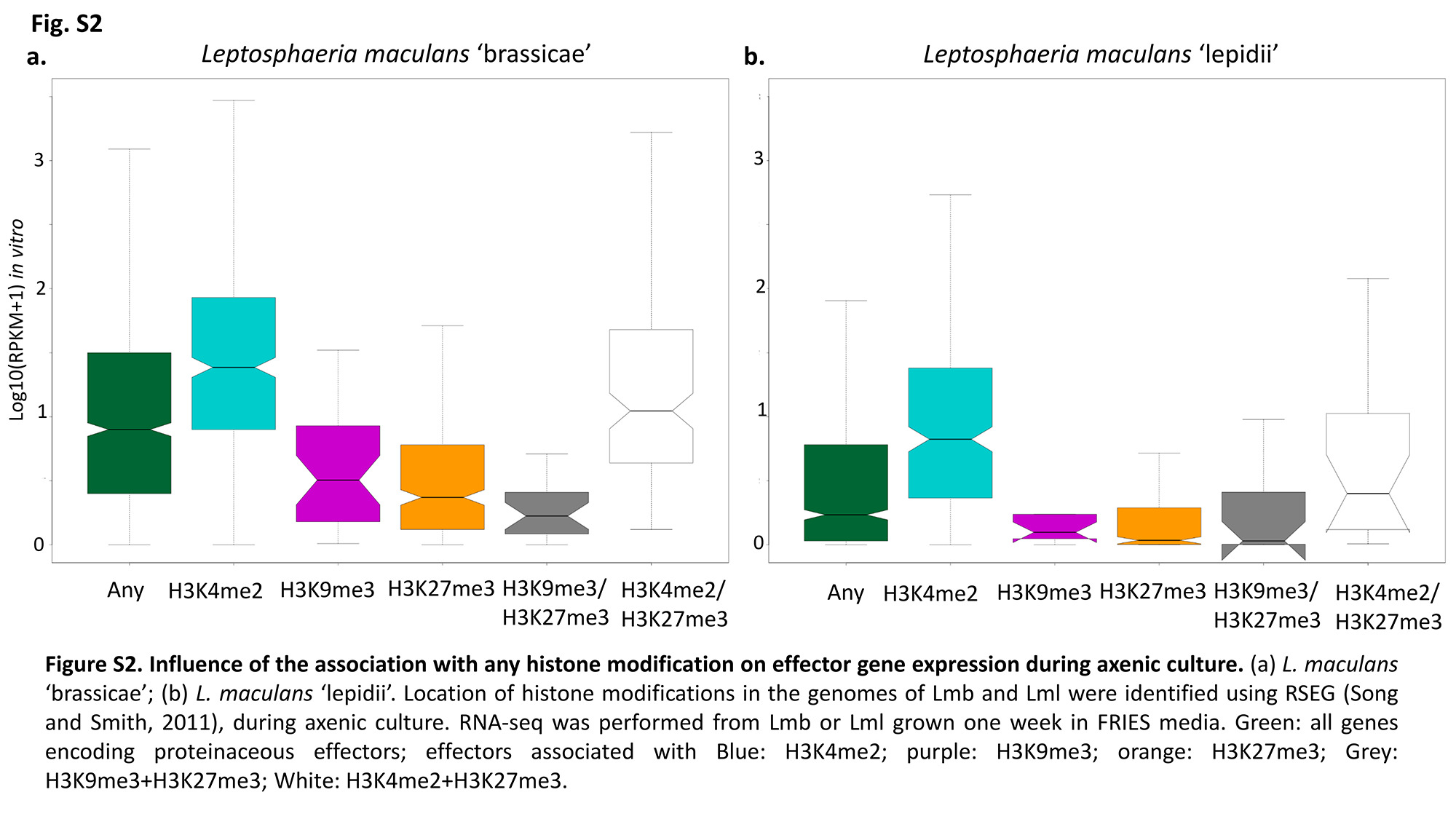

Supplement: Supplementary file 3 — Influence of the association with H3K4me2, H3K9me3, H3K27me3 on effector gene expression during axenic culture. (a) L. maculans ‘brassicae’, Lmb; (b) L. maculans ‘lepidii’, Lml. Location of histone modifications in the genomes of Lmb and Lml were identified using RSEG (Song and Smith 2011), during axenic culture. RNA-seq was performed from Lmb or Lml grown one week in FRIES media. Green: all genes encoding proteinaceous effectors; effectors associated with blue: H3K4me2; purple: H3K9me3; orange: H3K27me3; grey: H3K9me3+H3K27me3; white: H3K4me2+H3K27me3. (PNG 6596 kb) [file 10577_2021_9658_Fig8_ESM.png]

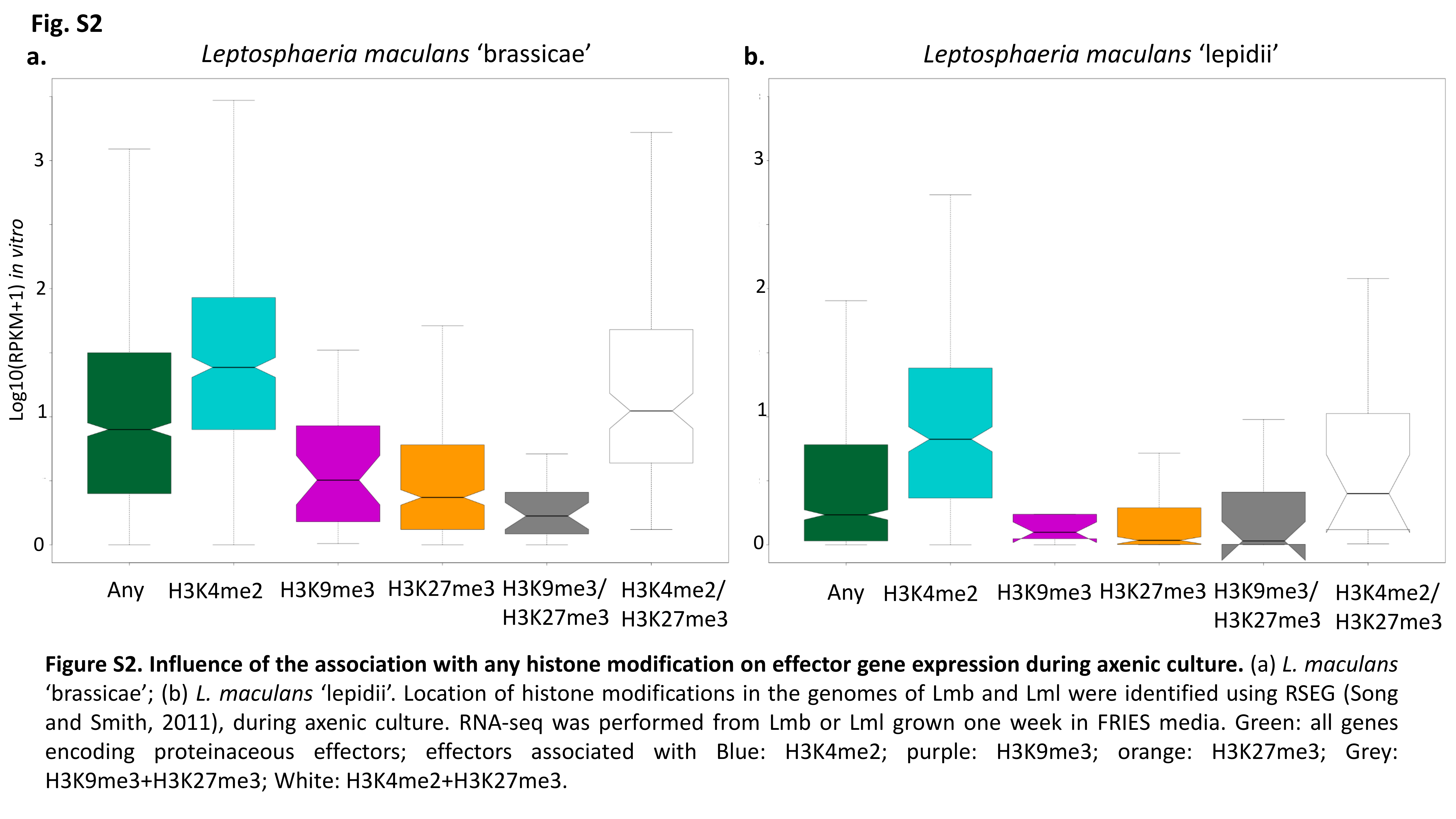

Supplement: Supplementary file 4 — High Resolution (TIF 1960 kb) [file 10577_2021_9658_MOESM2_ESM.tif]

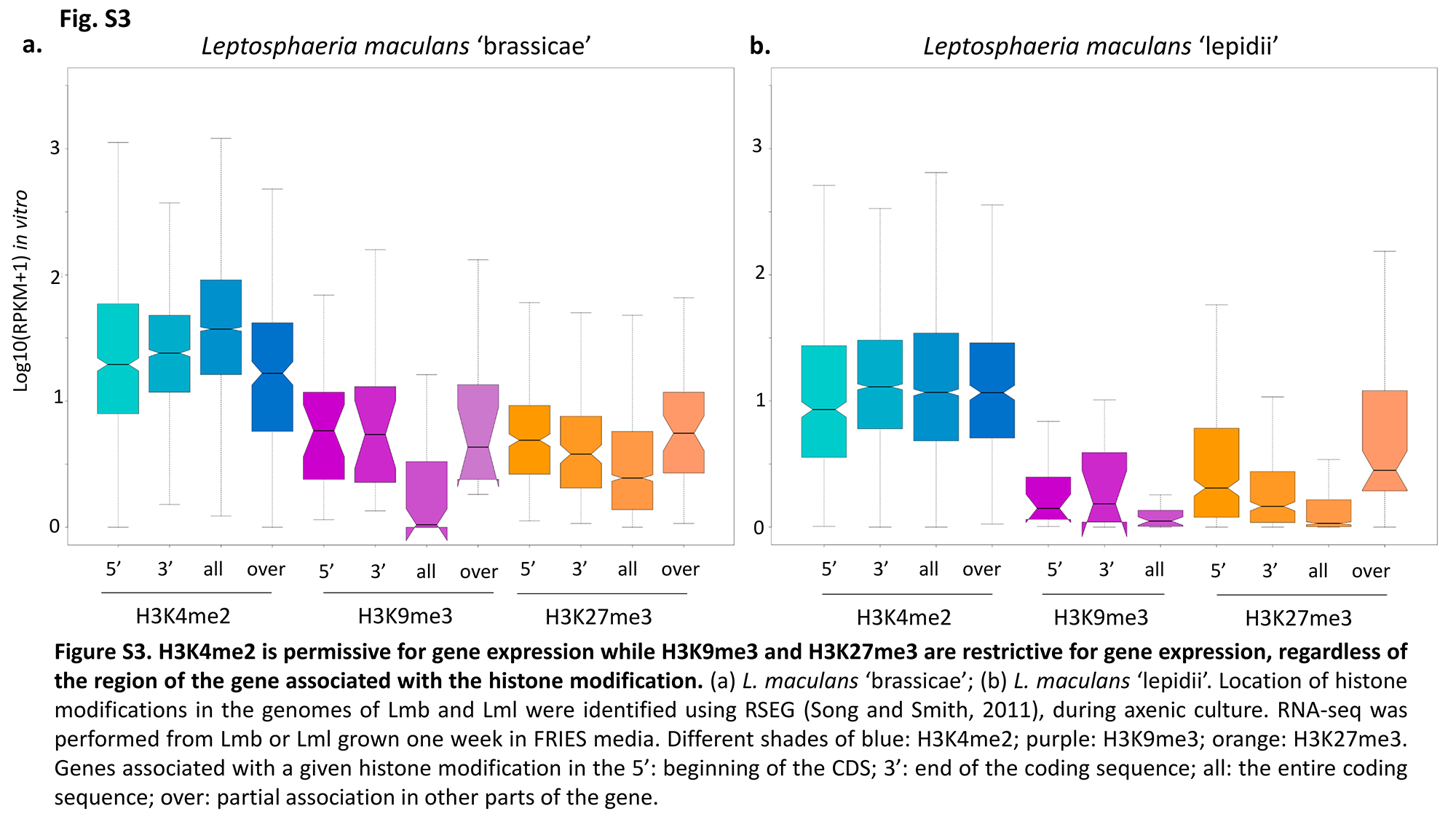

Supplement: Supplementary file 5 — H3K4me2 is permissive for gene expression while H3K9me3 and H3K27me3 are restrictive for gene expression, regardless of the region of the gene associated with the histone modification. (a) L. maculans ‘brassicae’, Lmb; (b) L. maculans ‘lepidii’, Lml. Location of histone modifications in the genomes of Lmb and Lml were identified using RSEG (Song and Smith 2011), during axenic culture. RNA-seq was performed from Lmb or Lml grown one week in FRIES media. Different shades of blue: H3K4me2; purple: H3K9me3; orange: H3K27me3. Genes associated with a given histone modification in the 5’: beginning of the CDS; 3’: end of the coding sequence; all: the entire coding sequence; over: partial association in other parts of the gene. (PNG 8794 kb) [file 10577_2021_9658_Fig9_ESM.png]

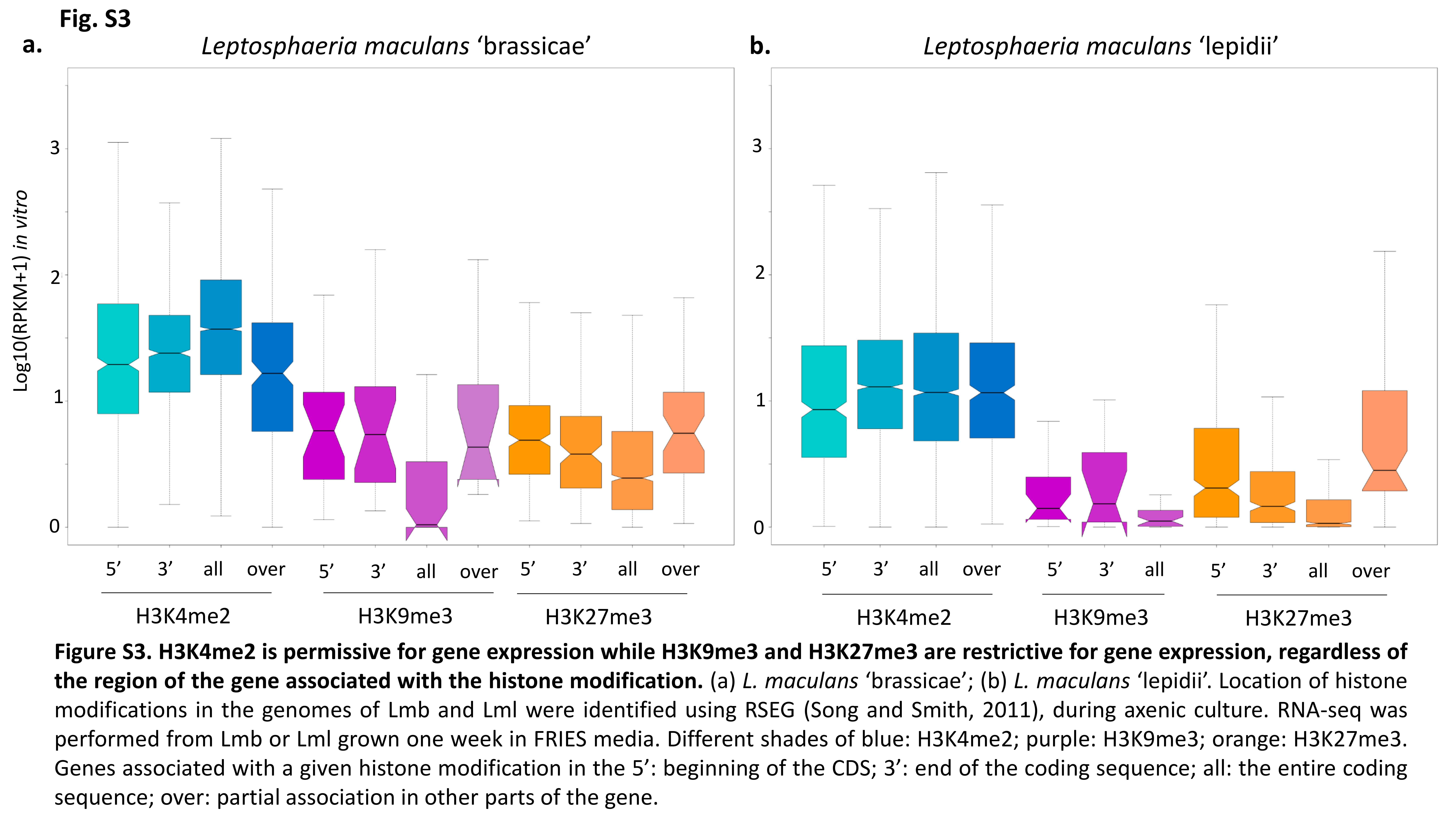

Supplement: Supplementary file 6 — High Resolution (TIF 2581 kb) [file 10577_2021_9658_MOESM3_ESM.tif]
