# Supplementary material for: Genome-wide mapping of histone modifications during axenic growth in two species of Leptosphaeria maculans showing contrasting genomic organization
Source: Chromosome Res. 2021 May 21;29(2):219–36. doi: 10.1007/s10577-021-09658-1 (PMC8159818; doi:10.1007/s10577-021-09658-1)
Supplement: Supplementary file 7 — Statistics of ChIP-seq and RNA-seq datasets and alignments. Table shows the number of reads used for the alignment, the number of reads mapping only at one location, the number of reads aligned more than once and the unmapped reads against the genome of L. maculans 'brassicae' (Lmb; Dutreux et al. 2018) or L. maculans 'lepidii' (Lml; Grandaubert et al. 2014). (DOCX 20.6 kb) [file 10577_2021_9658_MOESM4_ESM.docx]

|  | **Supplementary Table 1. Statistics of ChIP-seq and RNA-seq datasets and alignments** | | | | | | | | | | | |
| --- | --- | --- | --- | --- | --- | --- | --- | --- | --- | --- | --- | --- |
| Experiment | sample | ID | | Nb of reads | Nb not aligned | % not aligned | Nb aligned exactly one time | % aligned exactly one time | Nb aligned > 1 time | % aligned > 1 time | | overall alignement rate |
| ChIP-seq_Lmb | Lmb_A_K4 | | 1999_A | 9395841 | 158895 | 1.69 | 9009443 | 95.89 | 227503 | | 2.42 | 98.31 |
|  | Lmb_A_K9 | | 1999_B | 10919762 | 136322 | 1.25 | 1730751 | 15.85 | 9052689 | | 82.9 | 98.75 |
|  | Lmb_A_K27 | | 1999_C | 10529291 | 124737 | 1.18 | 9039896 | 85.85 | 1364658 | | 12.96 | 98.82 |
|  | Lmb_B_K4 | | 1999_D | 10534991 | 87375 | 0.83 | 10211829 | 96.93 | 235787 | | 2.24 | 99.17 |
|  | Lmb_B_K9 | | 1999_E | 10654189 | 85037 | 0.80 | 1472180 | 13.82 | 9096972 | | 85.38 | 99.2 |
|  | Lmb_B_K27 | | 1999_F | 10635233 | 56652 | 0.53 | 9449772 | 88.85 | 1128809 | | 10.61 | 99.47 |
|  | Lmb_C_K4 | | 1999_G | 10346671 | 84083 | 0.81 | 10076434 | 97.39 | 186154 | | 1.8 | 99.19 |
|  | Lmb_C_K9 | | 1999_H | 10459367 | 75082 | 0.72 | 1089336 | 10.41 | 9294949 | | 88.87 | 99.28 |
|  | Lmb_C_K27 | | 1999_I | 10710240 | 65549 | 0.61 | 9566489 | 89.32 | 1078202 | | 10.07 | 99.39 |
| ChIP-seq_Lml | Lml_A_K4 | | 1999_J | 11555456 | 514684 | 4.45 | 11014396 | 95.32 | 26376 | | 0.23 | 95.55 |
|  | Lml_A_K9 | | 1999_K | 9204550 | 1292960 | 14.05 | 5376256 | 58.41 | 2535334 | | 27.54 | 85.95 |
|  | Lml_A_K27 | | 1999_L | 10555063 | 993082 | 9.41 | 9370385 | 88.78 | 191596 | | 1.82 | 90.59 |
|  | Lml_B_K4 | | 1999_M | 10535483 | 519320 | 4.93 | 9999115 | 94.91 | 17048 | | 0.16 | 95.07 |
|  | Lml_B_K9 | | 1999_N | 10850527 | 1895009 | 17.46 | 6719265 | 61.93 | 2236253 | | 20.61 | 82.54 |
|  | Lml_B_K27 | | 1999_O | 10828748 | 983723 | 9.08 | 9680618 | 89.4 | 164407 | | 1.52 | 90.92 |
|  | Lml_C_K4 | | 1999_P | 10734950 | 358237 | 3.34 | 10364563 | 96.55 | 12150 | | 0.11 | 96.66 |
|  | Lml_C_K9 | | 1999_Q | 10875975 | 1278587 | 11.76 | 4634043 | 42.61 | 4963345 | | 45.64 | 88.24 |
|  | Lml_C_K27 | | 1999_R | 11402138 | 823586 | 7.22 | 10308659 | 90.41 | 269893 | | 2.37 | 92.78 |
| RNA-seq_Lml | Lml_A | | NDPS10 | 48390396 | 1310373 | 2.71 | 46986310 | 97.09 | 93713 | | 0.19 | 97.28 |
|  | Lml_B | | NDPS11 | 47495796 | 1238185 | 2.60 | 46141848 | 97.14 | 115763 | | 0.24 | 97.40 |
|  | Lml_C | | NDPS12 | 47562379 | 1294793 | 2.72 | 46174275 | 97.08 | 93311 | | 0.2 | 97.28 |
| RNA-seq_Lmb | Lmb_A | | NDPS13 | 43934637 | 726522 | 1.65 | 41770754 | 95.07 | 1437361 | | 3.27 | 98.34 |
|  | Lmb_B | | NDPS14 | 43634661 | 610333 | 1.39 | 42207134 | 96.72 | 817194 | | 1.87 | 98.6 |
|  | Lmb_C | | NDPS15 | 47642236 | 754749 | 1.58 | 45930697 | 96.4 | 956790 | | 2 | 98.41 |
| Table shows the number of reads used for the alignment, the number of reads mapping only at one location, number of reads aligned more than once and the unmapped reads against the genome of *L. maculans* 'brassicae' (Dutreux et al. 2018) or *L. maculans* 'lepidii' (Grandaubert et al. 2014). | | | | | | | | | | | | |
