# Supplementary material for: Genome-wide mapping of histone modifications during axenic growth in two species of Leptosphaeria maculans showing contrasting genomic organization
Source: Chromosome Res. 2021 May 21;29(2):219–36. doi: 10.1007/s10577-021-09658-1 (PMC8159818; doi:10.1007/s10577-021-09658-1)
Supplement: Supplementary file 9 — Correlation analysis between the different ChIP experiments generated with antibodies targeting histone modifications H3K4me2, H3K9me3 and H3K27me3 in Leptosphaeria maculans ‘lepidii’, during in vitro growth. Correlation analyses were performed to analyse location of the significantly enriched domains, identified using RSEG (Song and Smith 2011), from the three different biological replicates generated and analyzed separately. A Kendall's Ƭ correlation test was performed using R. (DOCX 18 kb) [file 10577_2021_9658_MOESM6_ESM.docx]

| **Supplementary Table 3. Correlation analysis between the different ChIP experiments generated with antibodies targeting histone modifications H3K4me2, H3K9me3 and H3K27me3 in *Leptosphaeria maculans* 'lepidii', during *in vitro* growth** | | | | | | | | | | | |
| --- | --- | --- | --- | --- | --- | --- | --- | --- | --- | --- | --- |
|  |  |  |  |  |  |  |  |  |  |  |  |
|  |  |  |  |  |  |  |  |  |  |  |  |
|  |  |  | H3K4me2 | | | H3K9me3 | | | H3K27me3 | | |
|  |  |  | 1999_J | 1999_M | 1999_P | 1999_K | 1999_N | 1999_Q | 1999_L | 1999_O | 1999_R |
|  | H3K4me2 | 1999_J | 1.00 |  |  |  |  |  |  |  |  |
|  |  | 1999_M | 0.94 | 1.00 |  |  |  |  |  |  |  |
|  |  | 1999_P | 0.89 | 0.90 | 1.00 |  |  |  |  |  |  |
|  | H3K9me3 | 1999_K | -0.21 | -0.21 | -0.23 | 1.00 |  |  |  |  |  |
|  |  | 1999_N | 0.22 | 0.23 | 0.24 | -0.88 | 1.00 |  |  |  |  |
|  |  | 1999_Q | -0.26 | -0.26 | -0.28 | 0.78 | -0.77 | 1.00 |  |  |  |
|  | H3K27me3 | 1999_L | -0.21 | -0.21 | -0.22 | -0.05 | 0.08 | 0.07 | 1.00 |  |  |
|  |  | 1999_O | -0.22 | -0.22 | -0.23 | -0.06 | 0.09 | 0.05 | 0.89 | 1.00 |  |
|  |  | 1999_R | -0.30 | -0.30 | -0.31 | -0.05 | 0.07 | 0.05 | 0.74 | 0.80 | 1.00 |
|  |  |  |  |  |  |  |  |  |  |  |  |
| Correlation analyses were performed to analyze location of the significantly enriched domains, identified using RSEG (Song and Smith 2011), from the three different biological replicates generated and analyzed separately. A Kendall's *Ƭ* correlation test was performed using R. | | | | | | | | | | | |
|  |  |  |  |  |  |  |  |  |  |  |  |
