# Supplementary material for: Genome-wide mapping of histone modifications during axenic growth in two species of Leptosphaeria maculans showing contrasting genomic organization
Source: Chromosome Res. 2021 May 21;29(2):219–36. doi: 10.1007/s10577-021-09658-1 (PMC8159818; doi:10.1007/s10577-021-09658-1)
Supplement: Supplementary file 10 — Coverage of histone modifications H3K4me2, H3K9me3 and H3K27me3 in the genome of Leptosphaeria maculans ‘brassicae’. aGenome as published in Dutreux et al. (2018). bLocation of H3K4me2, H3K9me3 and H3K27me3 was determined through ChIP-seq analysis, in vitro, and regions significantly enriched for any of the modifications was identified using RSEG (Song and Smith 2011). (DOCX 19 kb) [file 10577_2021_9658_MOESM7_ESM.docx]

| **Supplementary Table 4. Coverage of histone modifications H3K4me2, H3K9me3 and H3K27me3 in the genome of *Leptosphaeria maculans* 'brassicae'** | | | | | | | |
| --- | --- | --- | --- | --- | --- | --- | --- |
|  |  |  |  |  |  |  |  |
| ID_SC^a^ | SC size (bp)^a^ | H3K4me2^b^ | | H3K9me3^b^ | | H3K27me3^b^ | |
|  |  | bp | % | bp | % | bp | % |
| JN3_BAC01 | 21008 | 0 | 0.00 | 21000 | 99.96 | 1251 | 5.95 |
| JN3_SC00 | 3635781 | 1307171 | 35.95 | 1102622 | 30.33 | 762986 | 20.99 |
| JN3_SC01 | 3650661 | 1483193 | 40.63 | 1047969 | 28.71 | 687488 | 18.83 |
| JN3_SC02 | 3465289 | 1568755 | 45.27 | 821441 | 23.70 | 640962 | 18.50 |
| JN3_SC03 | 3116548 | 1193866 | 38.31 | 999254 | 32.06 | 574441 | 18.43 |
| JN3_SC04 | 3080038 | 1425610 | 46.29 | 665755 | 21.62 | 540171 | 17.54 |
| JN3_SC05 | 2723307 | 963377 | 35.38 | 963038 | 35.36 | 579405 | 21.28 |
| JN3_SC06 | 2669349 | 1086759 | 40.71 | 921773 | 34.53 | 488919 | 18.32 |
| JN3_SC07 | 2437616 | 1197276 | 49.12 | 522765 | 21.45 | 587443 | 24.10 |
| JN3_SC08 | 2329530 | 727752 | 31.24 | 1123307 | 48.22 | 306359 | 13.15 |
| JN3_SC09 | 2121901 | 748451 | 35.27 | 886766 | 41.79 | 379620 | 17.89 |
| JN3_SC10 | 1953324 | 918611 | 47.03 | 482165 | 24.68 | 439910 | 22.52 |
| JN3_SC11 | 1933728 | 1000894 | 51.76 | 400992 | 20.74 | 273848 | 14.16 |
| JN3_SC12 | 1809099 | 748253 | 41.36 | 486740 | 26.91 | 486379 | 26.89 |
| JN3_SC13 | 1787499 | 765814 | 42.84 | 565221 | 31.62 | 372365 | 20.83 |
| JN3_SC14 | 1545248 | 808577 | 52.33 | 318036 | 20.58 | 342119 | 22.14 |
| JN3_SC15 | 1485921 | 587315 | 39.53 | 451809 | 30.41 | 387118 | 26.05 |
| JN3_SC16 | 1416674 | 556218 | 39.26 | 545236 | 38.49 | 243093 | 17.16 |
| JN3_SC17 | 1391278 | 480336 | 34.52 | 588903 | 42.33 | 415859 | 29.89 |
| JN3_SC18 | 1351186 | 562433 | 41.63 | 490141 | 36.27 | 258335 | 19.12 |
| JN3_SC19 | 832888 | 44716 | 5.37 | 748487 | 89.87 | 56517 | 6.79 |
| JN3_SC20 | 252801 | 0 | 0.00 | 197121 | 77.97 | 32524 | 12.87 |
| JN3_SC21 | 237616 | 0 | 0.00 | 197118 | 82.96 | 18507 | 7.79 |
| JN3_SC22 | 206368 | 0 | 0.00 | 178511 | 86.50 | 4003 | 1.94 |
| JN3_SC23 | 157065 | 0 | 0.00 | 0 | 0.00 | 0 | 0.00 |
| JN3_SC24 | 81359 | 0 | 0.00 | 54108 | 66.51 | 751 | 0.92 |
| JN3_SC25 | 68927 | 0 | 0.00 | 65503 | 95.03 | 5502 | 7.98 |
| JN3_SC26 | 58575 | 0 | 0.00 | 57354 | 97.92 | 3254 | 5.56 |
| JN3_SC27 | 50646 | 0 | 0.00 | 26005 | 51.35 | 1502 | 2.97 |
| JN3_SC28 | 36829 | 0 | 0.00 | 34500 | 93.68 | 6753 | 18.34 |
| JN3_SC29 | 33591 | 0 | 0.00 | 22753 | 67.74 | 15503 | 46.15 |
| JN3_SC30 | 23239 | 0 | 0.00 | 20901 | 89.94 | 0 | 0.00 |
| JN3_SC31 | 21588 | 0 | 0.00 | 21001 | 97.28 | 0 | 0.00 |
| genome | 45986477 | 18175377 | 39.52 | 15028295 | 32.68 | 8912887 | 19.38 |
| ^a^Genome as published in Dutreux et al. (2018) | | | |  |  |  |  |
| ^b^Location of H3K4me2, H3K9me3 and H3K27me3 was determined through ChIP-seq analysis, *in vitro*, and regions significantly enriched for any of the modifications was identified using RSEG (Song and Smith 2011). | | | | | | | |
|  |  |  |  |  |  |  |  |
|  |  |  |  |  |  |  |  |
