# Supplementary material for: Genome-wide mapping of histone modifications during axenic growth in two species of Leptosphaeria maculans showing contrasting genomic organization
Source: Chromosome Res. 2021 May 21;29(2):219–36. doi: 10.1007/s10577-021-09658-1 (PMC8159818; doi:10.1007/s10577-021-09658-1)
Supplement: Supplementary file 12 — GO categories enriched in genes associated with H3K4me2 during axenic culture of Leptosphaeria maculans ‘brassicae’. GO annotation of the Lmb genes was retrieved from Dutreux et al. (2018). Analysis of GO enrichment among the genes associated with H3K4me2 during axenic culture of Lmb was performed using Cytoscape (Shannon et al. 2003). (DOCX 20.4 kb) [file 10577_2021_9658_MOESM9_ESM.docx]

| **Supplementary Table 6. GO categories enriched in genes associated with H3K4me2 during axenic culture of *Leptosphaeria maculans* 'brassicae'** | | | | | | | | |  |
| --- | --- | --- | --- | --- | --- | --- | --- | --- | --- |
| GO-ID | FDR | nb. of genes with the given GO term among the genes associated with H3K4me2 | nb. of genes with a GO annotation among the genes associated with H3K4me2 | proportion in the H3K4me2-genes | nb. of genes with the given GO in the genome | nb. of genes with a GO annotation in the genome | proportion in the genome | description |  |
| 9987 | 3.60E-13 | 1976 | 3276 | 6.03E-01 | 2700 | 5076 | 5.32E-01 | cellular_process |  |
| 44237 | 6.85E-06 | 1585 | 3276 | 4.84E-01 | 2203 | 5076 | 4.34E-01 | cellular_metabolic_process |  |
| 71840 | 6.85E-06 | 540 | 3276 | 1.65E-01 | 661 | 5076 | 1.30E-01 | cellular_component_organization_or_biogenesis |  |
| 10467 | 1.10E-05 | 498 | 3276 | 1.52E-01 | 606 | 5076 | 1.19E-01 | gene_expression |  |
| 1901576 | 4.13E-05 | 698 | 3276 | 2.13E-01 | 899 | 5076 | 1.77E-01 | organic_substance_biosynthetic_process |  |
| 44249 | 4.13E-05 | 690 | 3276 | 2.11E-01 | 888 | 5076 | 1.75E-01 | cellular_biosynthetic_process |  |
| 44085 | 4.76E-05 | 336 | 3276 | 1.03E-01 | 392 | 5076 | 7.72E-02 | cellular_component_biogenesis |  |
| 6807 | 1.06E-04 | 1498 | 3276 | 4.57E-01 | 2102 | 5076 | 4.14E-01 | nitrogen_compound_metabolic_process |  |
| 9058 | 1.06E-04 | 727 | 3276 | 2.22E-01 | 950 | 5076 | 1.87E-01 | biosynthetic_process |  |
| 34645 | 2.43E-04 | 398 | 3276 | 1.21E-01 | 487 | 5076 | 9.59E-02 | cellular_macromolecule_biosynthetic_process |  |
| 9059 | 4.92E-04 | 402 | 3276 | 1.23E-01 | 497 | 5076 | 9.79E-02 | macromolecule_biosynthetic_process |  |
| 44271 | 4.92E-04 | 475 | 3276 | 1.45E-01 | 600 | 5076 | 1.18E-01 | cellular_nitrogen_compound_biosynthetic_process |  |
| 44267 | 6.15E-04 | 562 | 3276 | 1.72E-01 | 726 | 5076 | 1.43E-01 | cellular_protein_metabolic_process |  |
| 44260 | 1.37E-03 | 834 | 3276 | 2.55E-01 | 1129 | 5076 | 2.22E-01 | cellular_macromolecule_metabolic_process |  |
| 1901566 | 1.44E-03 | 445 | 3276 | 1.36E-01 | 565 | 5076 | 1.11E-01 | nitrogen_compound_biosynthetic_process |  |
| 34641 | 1.48E-03 | 928 | 3276 | 2.83E-01 | 1270 | 5076 | 2.50E-01 | cellular_nitrogen_compound_metabolic_process |  |
| 33036 | 1.62E-03 | 229 | 3276 | 6.99E-02 | 266 | 5076 | 5.24E-02 | macromolecule_localization |  |
| 22613 | 3.68E-03 | 224 | 3276 | 6.84E-02 | 263 | 5076 | 5.18E-02 | ribonucleoprotein_complex_biogenesis |  |
| 16043 | 4.63E-03 | 343 | 3276 | 1.05E-01 | 429 | 5076 | 8.45E-02 | cellular_component_organization |  |
| 8104 | 4.90E-03 | 208 | 3276 | 6.35E-02 | 243 | 5076 | 4.79E-02 | protein_localization |  |
| 43604 | 5.27E-03 | 237 | 3276 | 7.23E-02 | 283 | 5076 | 5.58E-02 | amide_biosynthetic_process |  |
| 19538 | 5.81E-03 | 671 | 3276 | 2.05E-01 | 904 | 5076 | 1.78E-01 | protein_metabolic_process |  |
| 42254 | 6.77E-03 | 208 | 3276 | 6.35E-02 | 245 | 5076 | 4.83E-02 | ribosome_biogenesis |  |
| 43603 | 7.05E-03 | 266 | 3276 | 8.12E-02 | 325 | 5076 | 6.40E-02 | cellular_amide_metabolic__process |  |
| 51641 | 7.62E-03 | 224 | 3276 | 6.84E-02 | 268 | 5076 | 5.28E-02 | cellular_localization |  |
| 45184 | 7.62E-03 | 179 | 3276 | 5.46E-02 | 207 | 5076 | 4.08E-02 | establishment_of_protein_localization |  |
| 44238 | 7.62E-03 | 1652 | 3276 | 5.04E-01 | 2391 | 5076 | 4.71E-01 | primary_metabolic_process |  |
| 43043 | 7.62E-03 | 201 | 3276 | 6.14E-02 | 237 | 5076 | 4.67E-02 | peptide_biosynthetic_process |  |
| 15031 | 7.62E-03 | 175 | 3276 | 5.34E-02 | 202 | 5076 | 3.98E-02 | protein_transport |  |
| 71704 | 7.62E-03 | 1745 | 3276 | 5.33E-01 | 2536 | 5076 | 5.00E-01 | organic_substance_metabolic_process |  |
| 6518 | 7.62E-03 | 211 | 3276 | 6.44E-02 | 251 | 5076 | 4.94E-02 | peptide_metabolic_process |  |
| 6412 | 7.62E-03 | 197 | 3276 | 6.01E-02 | 232 | 5076 | 4.57E-02 | translation |  |
| 43170 | 9.05E-03 | 1129 | 3276 | 3.45E-01 | 1595 | 5076 | 3.14E-01 | macromolecule_metabolic_process |  |
| 42886 | 9.72E-03 | 182 | 3276 | 5.56E-02 | 213 | 5076 | 4.20E-02 | amide_transport |  |
|  |  |  |  |  |  |  |  |  |  |
| GO annotation of the Lmb genes was retrieved from Dutreux et al. (2018). Analysis of GO enrichment among the genes associated with H3K4me2 during axenic culture of Lmb was performed using Cytoscape (Shannon et al. 2003). | | | | | | | | | |
|  |  |  |  |  |  |  |  |  |  |
