# Supplementary material for: Genome-wide mapping of histone modifications during axenic growth in two species of Leptosphaeria maculans showing contrasting genomic organization
Source: Chromosome Res. 2021 May 21;29(2):219–36. doi: 10.1007/s10577-021-09658-1 (PMC8159818; doi:10.1007/s10577-021-09658-1)
Supplement: Supplementary file 15 — GO categories enriched in genes associated with H3K27me3 during axenic culture of Leptosphaeria maculans 'lepidii'. GO annotation of the Lml genes was retrieved from Grandaubert et al. (2014). Analysis of GO enrichment among the genes associated with H3K27me3 during axenic culture of Lml was performed using Cytoscape (Shannon et al. 2003). (DOCX 19.2 kb) [file 10577_2021_9658_MOESM12_ESM.docx]

| **Supplementary Table 9. GO categories enriched in genes associated with H3K27me3 during axenic culture of *Leptosphaeria maculans* 'lepidii'** | | | | | | | | | |
| --- | --- | --- | --- | --- | --- | --- | --- | --- | --- |
| GO-ID | FDR | nb. of genes with the GO term among the genes associated with H3K27me3 | nb. of genes with a GO annotation among the genes associated with H3K27me3 | proportion in the H3K27me3-genes | nb. of genes with the given GO in the genome | nb. of genes with a GO annotation in the genome | proportion in the genome | description | |
| 5975 | 0.0024441 | 41 | 288 | 0.14236111 | 250 | 3725 | 0.06711409 | carbohydrate metabolic process | |
| 42221 | 0.0024441 | 20 | 288 | 0.06944444 | 88 | 3725 | 0.02362416 | response to chemical | |
| 51591 | 0.0024441 | 10 | 288 | 0.03472222 | 25 | 3725 | 0.00671141 | response to cAMP | |
| 46683 | 0.0024441 | 10 | 288 | 0.03472222 | 25 | 3725 | 0.00671141 | response to organophosphorus | |
| 10033 | 0.0024441 | 11 | 288 | 0.03819444 | 31 | 3725 | 0.00832215 | response to organic substance | |
| 10243 | 0.0024441 | 10 | 288 | 0.03472222 | 26 | 3725 | 0.00697987 | response to organonitrogen compound | |
| 1901698 | 0.0024441 | 10 | 288 | 0.03472222 | 26 | 3725 | 0.00697987 | response to nitrogen compound | |
| 14070 | 0.0024441 | 10 | 288 | 0.03472222 | 26 | 3725 | 0.00697987 | response to organic cyclic compound | |
| 14074 | 0.0024441 | 10 | 288 | 0.03472222 | 26 | 3725 | 0.00697987 | response to purine-containing compound | |
|  |  |  |  |  |  |  |  |  |  |
| GO annotation of the Lml genes was retrieved from Grandaubert et al. (2014). Analysis of GO enrichment among the genes associated with H3K27me3 during axenic culture  of Lml was performed using Cytoscape (Shannon et al. 2003). | | | | | | | | | |
|  |  |  |  |  |  |  |  |  |  |
