# Supplementary material for: Genome-wide mapping of histone modifications during axenic growth in two species of Leptosphaeria maculans showing contrasting genomic organization
Source: Chromosome Res. 2021 May 21;29(2):219–36. doi: 10.1007/s10577-021-09658-1 (PMC8159818; doi:10.1007/s10577-021-09658-1)
Supplement: Supplementary file 16 — Analysis of the localization of genes conserved between Leptosphaeria maculans ‘brassicae’ and Leptosphaeria maculans ‘lepidii’, in relation to domains enriched with H3K4me2, H3K9me3, H3K27me3, H3K4me2/H3K27me3 and H3K9me3/H3K27me3 during axenic culture. aNumber of genes located in a H3K4me2-, H3K9me3-, H3K27me3-, H3K4me2/H3K27me3-, H3K9me3/H3K27me3-domain in Lmb and conserved in Lml; blocation of the conserved genes of Lml in a H3K4me2-, H3K9me3-, H3K27me3-, H3K4me2/H3K27me3-, H3K9me3/H3K27me3-domain. (DOCX 16 kb) [file 10577_2021_9658_MOESM13_ESM.docx]

**Supplementary Table 10. Analysis of the localization of genes conserved between *Leptosphaeria maculans* 'brassicae' and *Leptosphaeria maculans* 'lepidii', in relation to domains enriched in H3K4me2-, H3K9me3-, H3K27me3-, H3K4me2/H3K27me3- and H3K9me3/H3K27me3-domains during axenic culture.**

| *L. maculans* 'brassicae' | | *L. maculans* 'lepidii' | |
| --- | --- | --- | --- |
| domain | number^a^ | number | domain^b^ |
| H3K4me2 | 4892 | 4092 | H3K4me2 |
|  |  | 5 | H3K9me3 |
|  |  | 29 | H3K27me3 |
|  |  | 102 | H3K4me2/H3K27me3 |
|  |  | 0 | H3K9me3/H3K27me3 |
|  |  | 4228 | total |
| H3K9me3 | 16 | 1 | H3K9me3 |
|  |  | 4 | H3K27me3 |
|  |  | 1 | H3K9me3/H3K27me3 |
|  |  | 6 | total |
| H3K27me3 | 833 | 22 | H3K4me2 |
|  |  | 1 | H3K9me3 |
|  |  | 402 | H3K27me3 |
|  |  | 10 | H3K4me2/H3K27me3 |
|  |  | 13 | H3K9me3/H3K27me3 |
|  |  | 448 | total |
| H3K4me2/H3K27me3 | 1318 | 589 | H3K4me2 |
|  |  | 0 | H3K9me3 |
|  |  | 188 | H3K27me3 |
|  |  | 137 | H3K4me2/H3K27me3 |
|  |  | 1 | H3K9me3/H3K27me3 |
|  |  | 915 | total |
| H3K9me3/H3K27me3 | 36 | 1 | H3K4me2 |
|  |  | 0 | H3K9me3 |
|  |  | 21 | H3K27me3 |
|  |  | 0 | H3K4me2/H3K27me3 |
|  |  | 2 | H3K9me3/H3K27me3 |
|  |  | 24 | total |

^a^Number of genes located in a H3K4me2-, H3K9me3-, H3K27me3-, H3K4me2/H3K27me3-, H3K9me3/H3K27me3-domain in Lmb and conserved in Lml;

^b^location of the conserved genes of Lml in a H3K4me2-, H3K9me3-, H3K27me3-, H3K4me2/H3K27me3-, H3K9me3/H3K27me3-domain.
