# Supplementary material for: Genome-wide mapping of histone modifications during axenic growth in two species of Leptosphaeria maculans showing contrasting genomic organization
Source: Chromosome Res. 2021 May 21;29(2):219–36. doi: 10.1007/s10577-021-09658-1 (PMC8159818; doi:10.1007/s10577-021-09658-1)
Supplement: Supplementary file 17 — Analysis of the enrichment of effector- or specific effector-genes in TE-rich regions, H3K4me2-, H3K9me3-, H3K27me3-, H3K9me3/H3K27me3-domains in the genomes of Leptosphaeria maculans ‘brassicae’ or Leptosphaeria maculans ‘lepidii’. aGenes located up to 2 kb upstream or downstream of a transposable element sequence; bGenes located in a H3K4me2-, H3K9me3-, H3K27me3- or H3K9me3/H3K27me3-domain in vitro; cA X2 test was applied to compare proportion of effector genes in the genome and in the genomic compartment analyzed; dOdds ratio was reported as a measure of the effect size. (DOCX 18 kb) [file 10577_2021_9658_MOESM14_ESM.docx]

**Supplementary Table 11. Analysis of the enrichment of effector- or specific effector-genes in TE-rich regions, H3K4me2-, H3K9me3-, H3K27me3-, H3K9me3/H3K27me3-domains in the genomes of *Leptosphaeria maculans* 'brassicae' and *Leptosphaeria maculans* 'lepidii'**

|  | *L. maculans* 'brassicae' | | | | | | | *L. maculans* 'lepidii' | | | | | | |  |  |  |  |  |  |  |
| --- | --- | --- | --- | --- | --- | --- | --- | --- | --- | --- | --- | --- | --- | --- | --- | --- | --- | --- | --- | --- | --- |
|  |  | effector-genes | | | specific effector-genes | | |  | effector-genes | | | specific effector-genes | | |  |  |  |  |  |  |  |
|  | number of genes | number | *P value*^c^ | Odds ratio^d^ | number | *P value*^c^ | Odds ratio^d^ | number of genes | number | *P value*^c^ | Odds ratio^d^ | number | *P value*^c^ | Odds ratio^d^ |  |  |  |  |  |  |  |
| genome | 13,047 | 1,080 | - | - | 806 | - | - | 11,272 | 892 | - | - | 618 | - | - |  |  |  |  |  |  |  |
| TE-associated genes^a^ | 2,466 | 289 | 5.6.10^-10^ | 1.41 | 210 | 1.4.10^-6^ | 1.37 | 641 | 79 | 3.7.10^-8^ | 1.55 | 66 | 3.4.10^-11^ | 1.88 |  |  |  |  |  |  |  |
| H3K4me2-domains^b^ | 7,373 | 433 | 6.7.10^-14^ | 0.71 | 319 | 4.1.10^-11^ | 0.7 | 6,065 | 266 | 3.9.10^-14^ | 0.55 | 163 | 5.8.10^-14^ | 0.49 |  |  |  |  |  |  |  |
| H3K9me3-domains^b^ | 104 | 38 | 2.2.10^-16^ | 4.41 | 35 | 2.2.10^-16^ | 5.44 | 70 | 14 | 1.3.10^-5^ | 2.53 | 14 | 1.8.10^-9^ | 3.64 |  |  |  |  |  |  |  |
| H3K27me3-domains^b^ | 2,020 | 286 | 2.2.10^-16^ | 1.71 | 200 | 3.6.10^-12^ | 1.6 | 1,501 | 217 | 2.2.10^-16^ | 1.83 | 152 | 2.2.10^-16^ | 1.85 |  |  |  |  |  |  |  |
| H3K9me3+  H3K27me3-domains^b^ | 101 | 24 | 1.6.10^-8^ | 2.87 | 16 | 5.5.10^-5^ | 2.56 | 58 | 18 | 2.8.10^-13^ | 3.92 | 14 | 2.2.10^-16^ | 18.22 |  |  |  |  |  |  |  |
| ^a^Genes located up to 2 kb upstream or downstream of a transposable element sequence; | | | | | | | | | | | | | | | | | | | | | |
| ^b^Genes located in a H3K4me2-, H3K9me3-, H3K27me3- or H3K9me3/H3K27me3-domain *in vitro*; | | | | | | | | | | | | | | | | | | | | | |
| ^c^A X² test was applied to compare proportion of effector genes in the genome and in the genomic compartment analysed; | | | | | | | | | | | | | | | | | | | | | |
| ^d^Odds ratio was reported as a measure of the effect size. | | | | | | | | | | | | | | | |  |  |  |  |  |  |
