# Supplementary material for: Genome-wide mapping of histone modifications during axenic growth in two species of Leptosphaeria maculans showing contrasting genomic organization
Source: Chromosome Res. 2021 May 21;29(2):219–36. doi: 10.1007/s10577-021-09658-1 (PMC8159818; doi:10.1007/s10577-021-09658-1)
Supplement: Supplementary file 18 — Location of (putative) metabolic effector in Leptosphaeria maculans ‘brassicae’ and Leptosphaeria maculans ‘lepidii’ genomic compartments. aGenes located up to 2 kb upstream or downstream of a transposable element sequence; bGenes with RPKM≥ 2; cGenes located in a H3K4me2-, H3K9me3-, H3K27me3- or H3K4me2/H3K27me3-domain in vitro; K4: H3K4me2; K9: H3K9me3; K27:H3K27me3. (DOCX 20.8 kb) [file 10577_2021_9658_MOESM15_ESM.docx]

| **Supplementary Table 12. Location of (putative) metabolic effector in *Leptosphaeria maculans* 'brassicae' and *Leptosphaeria maculans* 'lepidii' genomic compartments** | | | | | | | | | | |
| --- | --- | --- | --- | --- | --- | --- | --- | --- | --- | --- |
| **type** | **Gene ID** | **Secondary metabolite produced** | **Localization close to a TE in the Lmb genome^a^ (Yes / No)** | **Expressed during axenic growth of Lmb (Yes/No)^b^** | **Associated histone modification in Lmb^c^** | **Orthologous gene in Lml** | **Identity compared to Lmb (%)** | **Localization close to aTE in Lml genome^a^ (Yes / No)** | **Expressed during axenic growth of Lml (Yes/No)** | **Associated histone modification in Lml^c^** |
| **NRPS** | Lm_jn3_00988 | Sirodesmin | no | yes | K4/K27 | Lm_ibcn84_P008587 | 90% | no | yes | K27 |
|  | Lm_jn3_09218 | - | no | yes | K4 | Lm_ibcn84_P004314 | 98% | no | yes | N/A |
|  | Lm_jn3_03490 | - | no | yes | K27 | Lm_ibcn84_P001896 | 99% | no | no | K27 |
|  | Lm_jn3_02066 | Siderophore | no | yes | K4/K27 | Lm_ibcn84_P011015 | 90% | no | yes | K4 |
|  | Lm_jn3_10429 | - | no | yes | K4 | Lm_ibcn84_P005647 | 91% | no | yes | N/A |
|  | Lm_jn3_06945 | - | no | yes | K4/K27 | Lm_ibcn84_P001325 | 94% | no | yes | K4 |
|  | Lm_jn3_05352 | - | yes | yes | K4 | Lm_ibcn84_P004994 | 64% | no | yes | K4 |
|  | Lm_jn3_10094 | Siderophore | no | yes | K4/K27 | Lm_ibcn84_P005868 | 92% | no | yes | N/A |
|  | Lm_jn3_10827 | - | no | no | K27 | Lm_ibcn84_P005267 | 92% | no | no | K27 |
|  | Lm_jn3_02092 | Phomalide | yes | yes | 3 modifications | No match | - | - | - | - |
|  | Lm_jn3_08572 | Lysine | no | yes | K4/K27 | Lm_ibcn84_P010114 | 95% | no | yes | K4 |
|  | Lm_jn3_11822 | - | no | yes | K4/K27 | Lm_ibcn84_P011137 | 93% | no | yes | N/A |
|  | Lm_jn3_06609 | - | no | yes | K4 | Lm_ibcn84_P001023 | 94% | no | yes | N/A |
|  | Lm_jn3_10603 | - | no | yes | K27 | Lm_ibcn84_P005485 | 94% | no | yes | N/A |
| **PKS** | Lm_jn3_00998 | - | no | yes | K27 | No match | - | - | - | - |
|  | Lm_jn3_05348 | Phomenoic acid | no | yes | K4/K27 | Lm_ibcn84_P004990 | 96% | no | no | K27 |
|  | No match | - |  |  |  | Lm_ibcn84_P001547 | - | no | yes | K27 |
|  | Lm_jn3_02584 | - | no | yes | K27 | Lm_ibcn84_P000558 | 91% | yes | no | K27 |
|  | Lm_jn3_11562 | ABA | yes | no | K27 | No match | - | - | - | - |
|  | Lm_jn3_07864 | - | yes | yes | 3 modifications | Lm_ibcn84_P010582 | 87% | no | yes | K4 |
|  | Lm_jn3_12570 | - | no | yes | K27 | Lm_ibcn84_P010688 | 91% | no | yes | K27 |
|  | Lm_jn3_11296 | - | no | yes | K4 | Lm_ibcn84_P009766 | 67% | no | no | N/A |
|  | No match | - | - | - | - | Lm_ibcn84_P009768 | - | no | yes | K4 |
|  | Lm_jn3_11345 | Melanin | no | yes | K27 | Lm_ibcn84_P009804 | 98% | no | yes | N/A |
|  | Lm_jn3_06412 | - | no | yes | K27 | Lm_ibcn84_P004141 | 96% | no | yes | K27 |
|  | Lm_jn3_05162 | - | no | no | K27 | Lm_ibcn84_P004838 | 92% | no | no | K27 |
|  | Lm_jn3_01181 | - | no | yes | K27 | Lm_ibcn84_P008173 | 91% | no | yes | K27 |
|  | Lm_jn3_01173 | - | no | yes | K27 | Lm_ibcn84_P008166 | 96% | no | yes | K4/K27 |
|  | Lm_jn3_04807 |  | no | no | K27 | Lm_ibcn84_P008813 | 93% | no | no | K27 |
| ^a^Genes located up to 2 kb upstream or downstream of a transposable element sequence; | | | | | | |  |  |  |  |
| ^b^Genes with RPKM≥ 2; | | | | | |  |  |  |  |  |
| ^c^Genes located in a H3K4me2-, H3K9me3-, H3K27me3- or H3K4me2/H3K27me3-domain *in vitro;* K4: H3K4me2; K9: H3K9me3; K27:H3K27me3. | | | | | | | | | |  |
